# Regional and network neural activity reflect men’s preference for greater socioeconomic status during impression formation

**DOI:** 10.1038/s41598-020-76847-z

**Published:** 2020-11-20

**Authors:** Denise M. Barth, Bradley D. Mattan, Tzipporah P. Dang, Jasmin Cloutier

**Affiliations:** 1 Department of Psychological and Brain Sciences, University of Delaware, 105 The Green, Newark, DE 19716 USA; 2 Annenberg School for Communication, University of Pennsylvania, 3620 Walnut St., Philadelphia, PA 19104 USA

**Keywords:** Social neuroscience, Human behaviour, Perception

## Abstract

Evidence from social psychology suggests that men compared to women more readily display and pursue control over human resources or capital. However, studying how status and gender shape deliberate impression formation is difficult due to social desirability concerns. Using univariate and multivariate fMRI analyses (*n* = 65), we examined how gender and socioeconomic status (SES) may influence brain responses during deliberate but private impression formation. Men more than women showed greater activity in the VMPFC and NAcc when forming impressions of high-SES (vs. low-SES) targets. Seed partial least squares (PLS) analysis showed that this SES-based increase in VMPFC activity was associated with greater co-activation across an evaluative network for the high-SES versus low-SES univariate comparison. A data-driven task PLS analysis also showed greater co-activation in an extended network consisting of regions involved in salience detection, attention, and task engagement as a function of increasing target SES. This co-activating network was most pronounced for men. These findings provide evidence that high-SES targets elicit neural responses indicative of positivity, reward, and salience during impression formation among men. Contributions to a network neuroscience understanding of status perception and implications for gender- and status-based impression formation are discussed.

## Introduction

Social hierarchies are an intrinsic and systemic part of human culture^1^ and organizations^2^—ultimately helping to shape our motives^3^, affect^4^, and evaluations of the self^5^ and others^6–8^. The possession of high status is generally considered a positive characteristic^5,7,8^. However, also entrenched in society are the norms and roles associated with gender. Namely, women are presumed to occupy roles perceived to convey lower status in comparison to men^9^. These gendered social roles have long been known to shape how we attend to^10^ and evaluate others^9,11^, ultimately making it more difficult for women to attain higher status and leadership positions^12,13^. The perceiver’s own gender may also influence the degree to which status shapes evaluations of others^14^. Numerous studies have found that men tend to value acquiring and maximizing wealth more than women^15,16^, and some have argued that men compared to women more readily display and pursue control over human resources or capital^10,17,18^. In contrast, others have suggested that women may show a greater preference for high-status targets than do men, at least when it comes to long-term heterosexual mating partners^19,20^. In the present study, we were interested in testing which of these accounts (viz., gender roles or mate selection) might best explain neural responses to target status during impression formation. We therefore used fMRI to examine how target gender and socioeconomic status (SES) may influence brain responses during deliberate but private impression formation and how these responses may depend on the perceiver’s own gender.

### Regions of interest supporting status-based person evaluation

Based on recent work identifying the neural substrates of status-based evaluations^21–25^, we posit several key brains regions that may index status-based evaluation. In the domain of person evaluation, VMPFC responses have been shown to reflect increasingly positive evaluations based on available person knowledge^26,27^, including when such knowledge is about social status^6,21,23,28^. Beyond the VMPFC, a number of studies have shown sensitivity to status in regions implicated in the processing of reward and salience^6^. In general, the ventral striatum is thought to be sensitive to reward value and/or social salience^29^. In the domain of status perception, the ventral striatum appears to preferentially respond to high versus low status based on competence^30^ and positive versus negative outcomes in competitive scenarios^31^. However, other work suggests that responses to high status in this region are sensitive to a number of factors including the perceiver’s own status level^32^, culture^33^, and motivations^21^. Additionally, the amygdala, a region thought to be broadly involved in the assessment of social or biological relevance^34^ may be especially involved in the learning, updating, and recall of knowledge about status hierarchies^30,31,35^.

In line with the evidence reviewed above, we anticipated that the VMPFC, ventral striatum, and the amygdala would all tend to show greater responses to high-status compared to low-status targets, consistent with more positive evaluations of high-SES people^6^. We also examined which of two perceiver gender dynamics would impact impression formation of men and women varying in status. Based on evidence suggesting that men compared to women more readily value, display, and pursue control over human resources or capital^10,17,18^, men (vs. women) could show greater sensitivity to high- versus low-status individuals in brain regions and networks previously implicated in status-based evaluations. Alternatively, based on evidence of gendered asymmetry in the preference for status as a function of the perceiver’s and target’s gender during mate selection^19,20^, another possibility was that women more than men would show greater sensitivity to high status, but only for individuals of the other gender^cf.^^36^.

### Network approach to evaluations based on status and gender

In recent years, neuroimaging studies have increasingly adopted a network-based approach for characterizing the neural mechanisms underlying numerous cognitive phenomena^37^, including the domain of person perception^22,38^. This approach is important because distributed patterns of neural activity may not only account for more variance, but they may also better predict real-world outcomes^39^. At present, there are few studies that explore how networks of brain regions support status-based impression formation^22^. However, such approaches can be useful as a complement to focused ROI analyses, providing further insight into differences within and between participant groups. Complementing focused univariate ROI analyses, multivariate network analyses of fMRI data can offer a novel data-driven opportunity to discover meaningful associations between distributed patterns of brain activity and experimental conditions. In the present study, we used partial least squares (PLS)^40^ as a data-driven multivariate approach to identify patterns of coordinated neural activity associated with each combination of target gender and status. Although exploratory, we were particularly interested in whether functional connectivity would be sensitive to target status. Based on a review of the literature, we have previously focused on brain networks involved in status-based attention and evaluation^6^ and would expect greater co-activation in one or both of these networks in response to high-SES individuals, who are generally evaluated positively^6–8,14^. Accordingly, PLS allowed an initial exploration of this question.

## Methods

### Participants

Participants were recruited from the Chicago area via ads posted online, on public transportation, and fliers. In order to participate in the study, participants were required to meet the following criteria: (1) identify as White; (2) between 18 and 35 years old; (3) right-handed; (4) lived in the U.S. for at least 5 years; (5) good command of the English language; (6) no history of drug abuse; (7) no history of serious head injury; (8) no color vision problems; (9) no current acute illness; (10) not currently taking psychotropic medication; (11) no diagnosis of developmental disorders; (12) no diagnosis of a chronic disease that compromises mental, neural, or autonomic function; and (13) pass a standard MRI safety screen. Two-hundred fifteen people completed the initial screening procedures (see Supplemental Material S1). Of these 215 individuals, 69 eligible participants completed all parts of the study. Scanner data from three participants were corrupted during MR image reconstruction and therefore could not be used. One participant with an extremely low recorded response rate (9%) on the impression formation task indicated a lack of diligence and was removed as a sample outlier (i.e., exceeding 3 standard deviations from the sample mean recorded response rate: *M* = 93.75%, *SD* = 16.89%). This resulted in a final sample size of 65 participants (*M*_*Age*_ = 23.844, *SD*_*Age*_ = 4.334). Although a larger number of men (*n* = 37) than women (*n* = 28) were included in our final sample, the difference was non-significant, *X*^*2*^(1, *n* = 65) = 1.246, *p* = 0.264. Of the 63 participants who provided subjective status scores, women trended lower in subjective status (*M* = 6.571, *SD* = 1.665) than men (*M* = 7.257, *SD* = 1.482), but this difference was non-significant (see Supplemental Text S4 for full statistics).

#### Ethical guidelines and informed consent

This research was approved by the University of Chicago Social and Behavioral Sciences Institutional Review Board (IRB14-1232). All research was conducted in line with the University of Chicago’s ethical guidelines. All participants provided informed consent prior to participation.

### Protocol

Procedures and measures are outlined below. Further details on procedures and measures are provided in Supplemental Texts S1 and S2, respectively.

#### Gender identification and online pre-testing session

Upon passing the initial prescreen, participants were instructed to complete a series of surveys prior to arriving for their scanning appointment. In addition to reporting their gender (“Male”, “Female”, or “Other”), participants also completed several exploratory measures in this online pre-testing session (see Supplemental Text S2 for a full listing). No one in the analyzed sample indicated “Other” as a gender identity.

It bears mentioning that participants were asked to report their gender, which is a social construct, but the provided response options were for biological sex (male, female) instead of gender (man, woman). Base rates for these attributes are extremely correlated. Accordingly, we conflate sex and gender in this report. Although differentiating the effects of sex from effects of gender is beyond the scope of the present work, we acknowledge this as important future direction.

#### fMRI session

Eligible participants were scheduled for a two-and-a-half-hour appointment at the imaging center. Approximately thirty minutes of the appointment was spent on the pre-scan procedure (see Supplemental Text S2). The next hour of this session involved time in the scanner, and the last hour was spent completing the post-scan surveys and tasks (see Supplemental Text S2).

#### Arrival and preliminary measures

Participants first read and signed consent forms. After completing a brief survey (see Supplemental Text S2), participants received instructions regarding the different tasks. Participants learned that they would be completing two in-scanner tasks: one involving passively watching videos of police arrests (see Supplemental Text S1) and the other involving forming impressions of faces. Prior to the scanning session, participants learned to use the button boxes to indicate they formed their impression of each target (see below for details).

#### Scanner set-up and preliminary scans

Upon entering the scanner, participants were fitted with a button box on each hand. Once adequately situated in the scanner, participants completed two functional runs of an unrelated fMRI task (~ 13 min in total). This task involved viewing videos of police officers making arrests of civilians (see Supplemental Text S1 for details).

#### Status–color association training

Before completing the impression formation task, participants completed a self-paced status–color association training lasting approximately 10 min. Participants were told they would be forming impressions of individuals that vary in status, defined as SES^8^: “Those who have the highest social status tend to have the most money, the most education, and the most respected jobs. Those who have the lowest social status tend to have the least money, the least education, and the least respected jobs or no job”.

Because some perceptual antecedents (e.g., strength, height) are thought to convey status more readily for men than for women^41^, we opted to convey status information via knowledge-based cues. To this end, we trained participants to associate high and low SES with the colors blue or orange (see Supplemental Text S2)^21–23,42^. Status–color associations were counterbalanced across participants such that, for about half of our participants, blue represented low status and orange represented high status, and vice versa for the remainder of participants. Participants categorized: (1) blue and orange silhouettes as either high or low status (*M* = 95.57% for all trials); and (2) the words “high status” and “low status” as represented by either blue or orange (*M* = 90.14% for all trials). Participants had an average of 92.87% accuracy across these two training components. Finally, participants practiced forming impressions of two unique faces per gender that were not used in the experimental trials, each framed with blue or orange. Before proceeding to the fMRI impression formation task, participants had to report the correct status–color association back to the experimenter when prompted. Only one participant incorrectly recalled the status-color associations prior to the start of the first task run. The researcher corrected the participant and asked the participant to repeat back what they just learned. This participant also correctly recalled the associations prior to the second run.

#### Impression-formation fMRI task

**Figure 1:**
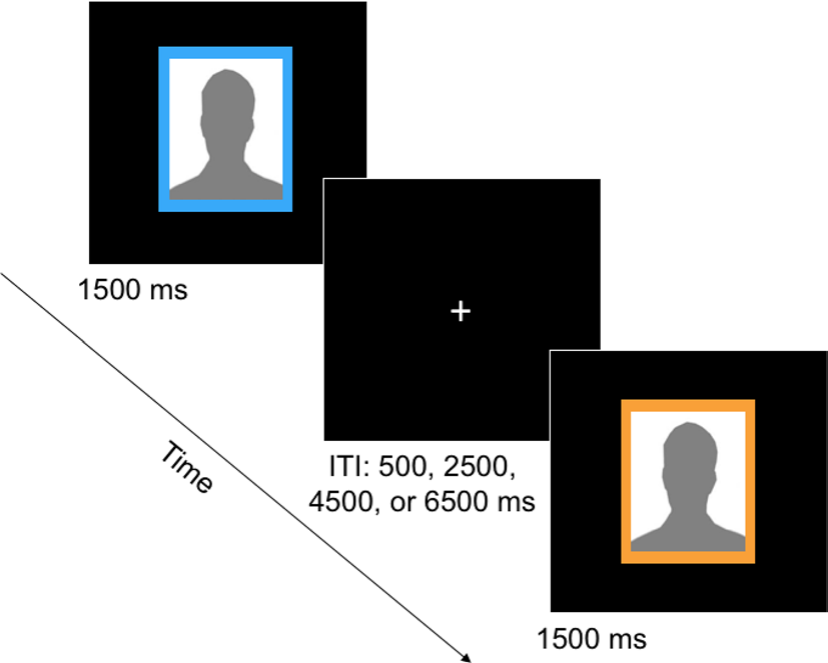
Trial procedure for impression formation fMRI task. A high (or low) SES woman’s (or man’s) face would appear (in place of the gray silhouettes pictured above) for 1500 ms followed by an intertrial interval (ITI) consisting of a fixation cross of a jittered duration between 500 and 6500 ms before the next face would appear. To indicate that they had formed their impressions for each face, participants responded by pressing simultaneously buttons assigned to each index finger. Participants were encouraged to finish forming their impressions by the time the face disappeared or shortly thereafter. Participants completed 112 total impression formation trials over two runs.

Having learned to associate different colors with different levels of status, participants then completed two functional runs of the impression formation task, each lasting approximately 7 min. Prior to the start of this fMRI task, participants learned that they would simply be forming overall impressions of faces. We guided participants further by indicating that there were no correct impressions. For each face, participants were asked to focus on their subjective thoughts and feelings based on all the information available. When they had formed an impression, they were instructed to respond by pressing both pointer fingers at the same time^21,23^. Each face was displayed for 1,500 ms, followed by a fixation cross with a jittered duration of 500, 2500, 4500, or 6500 ms (see Fig. 1). Participants were encouraged to form their impression prior to or shortly after the disappearance of each face stimulus. After entering timely responses during four practice trials (prior to initiating the scanner), participants began the first of two runs (98 TRs each). After the first run, the experimenter checked in with the participant and confirmed that they remembered the status-color associations. Once the participant correctly recalled the status–color associations, the experimenter reiterated the initial instructions and the second run began.

##### Stimuli

During each run, participants formed impressions of 28 unique faces distributed across the four gender–status groupings. These faces were equated on attractiveness, likeability, emotional intensity, actual age, and perceived age. We selected faces that were predominantly categorized as their documented gender (> 90% accuracy). However, men’s faces were rated with greater confidence as members of their documented gender and as more gender typical than were women’s faces (see Supplemental Text S3 for stimulus equating). Because these dimensions are less directly relevant to perceived status than other attributes such as likeability, age, and attractiveness, they were not prioritized during stimulus equating. Each unique face was presented twice per run. Each run corresponded to one of two unique fMRI-optimized trial sequences generated in optseq2^43^. The selected sequences met the constraint that they did not present more than three consecutive repetitions of a given condition.

##### Paradigm rationale and prior validation

We chose an unconstrained impression formation task because it allows participants to form their impressions anonymously based on all available information, thereby minimizing pressures to conform to any perceived expectations from the experimenter or society more generally^22^. Although this approach may minimize socially desirable responding, it also limits the experimenter’s ability to verify that participants are indeed forming impressions. Fortunately, a body of work using similar impression formation tasks has repeatedly found them to elicit activity in brain regions supporting social cognition (e.g., MPFC) when contrasted with non-social tasks^44,45^. More recent work using this impression formation task to focus specifically on status-based evaluative responses has consistently identified preferential VMPFC responses to high (vs. low) status along several social dimensions including moral status^23,24^ and SES^22^. These responses are consistent with other neuroimaging work focusing on status-based evaluations, which generally elicit greater VMPFC responses when participants evaluate high-status (vs. low-status) individuals in an array of paradigms beyond impression formation^6^.

#### Additional scans

After the final functional run, we acquired a resting state scan and an anatomical (MP-RAGE) scan over approximately 12 min, time permitting. The entire scanning session, including the unrelated fMRI task, was just under one hour long.

#### Post-scan measures and procedure

Participants exited the scanner and returned to the separate testing room to complete a series of exploratory measures and behavioral tasks. These post-scan tasks included exploratory measures of stimulus likeability and status recall for all face stimuli presented during the two scan runs (see Supplemental Text S1). After completion, participants were debriefed, paid, and excused.

### fMRI acquisition, pre-processing, and GLM

We used a Phillips dStream Achieva 3 T system and 32-channel head coil to acquire BOLD, T2* contrast-weighted EPIs. With a 2000-ms repetition time (TR) and a 25-ms echo time (TE), we acquired 34 oblique slices using an interleaved z-shim acquisition protocol^46^. Slices were 4-mm thick with a 0.5-mm gap, a 3 mm^2^ in-plane resolution, 77° flip angle, and a 192 × 134 × 192-mm field of view. Slices were aligned to the AC–PC axis of each participant^47^.

As in prior work^21^, EPIs from each participant’s two runs were pre-processed and analyzed using SPM8 and SPM8w (for details, see “Data and code availability”). We first implemented slice-time correction^48^, using the 17th slice acquisition as the reference. Subsequently, we integrated the four repeated z-shim slices^46^. The resulting images from each participant were then unwarped and realigned to the participant’s mean EPI to correct for motion and motion-by-distortion interactions^49^. Images were subsequently normalized to the MNI template and smoothed with an 8-mm FWHM kernel^50^.

To estimate the BOLD responses for each condition, each trial was considered as an event, and the stimulus time series was convolved with the canonical hemodynamic response function. A GLM modeled both scan sequences concatenated as a single session with regressors for each of the four conditions (ordered as follows: high-status woman, high-status man, low-status woman, and low-status man) followed by a standard set of regressors controlling for variance associated with various sources of systematic noise^21^. Contrast images reflecting the first-level effects of interest were used in second-level analyses.

### Analyses

The present analyses focused on potential effects of target gender and SES during impression formation and how these effects may be sensitive to the perceiver’s own gender. Our primary analyses focused on key regions of interest (ROI) identified above: the VMPFC, NAcc, and the amygdala. We also conducted exploratory analyses testing for any potential main effects of or interactions with the perceiver’s self-reported subjective SES. Including effects of subjective status in our ROI analyses did not change the results reported here, nor did any significant effects of subjective status emerge. For full results from all supplemental analyses, see Supplemental Text S4.

In an attempt to connect activity within our primary ROIs with co-activating regions throughout the brain, we followed up on the ROI analyses with an exploratory seed PLS analysis that focused on the main effect of perceived SES in the VMPFC. We also conducted a data-driven task PLS analysis to detect coordinated activity across the brain as a function of target gender and SES, unconstrained by any particular contrast. Finally, to further probe for effects of perceiver gender, we also examined the contribution of participant gender to the network identified by the task PLS analysis.

#### ROI analyses

Referencing prior neuroimaging work on status-based evaluation, we extracted BOLD activity from ROIs in the VMPFC, *MNI*_*x,y,z*_ = [0,52,− 6]^21,24,26^, left NAcc, *MNI*_*x,y,z*_ = [− 9,8,− 8] and right NAcc, *MNI*_*x,y,z*_ = [9,14,− 6]^21,51^, and left amygdala, *MNI*_*x,y,z*_ = [− 24,− 3,− 12] and right amygdala, *MNI*_*x,y,z*_ = [24,− 3,− 21]^21,52^. Average parameter estimates (vs. average signal response) were extracted for each condition from an 8-mm sphere (VMPFC) or a 4-mm sphere (NAcc, amygdala). The size of these ROIs is commensurate with differences in anatomical size between these regions and with ROI volumes from our previous work^21^.

ROIs were analyzed using the lme4 package for linear mixed-effects models^53^ in R^54^. Degrees of freedom were estimated using Satterthwaite’s approximation, provided by the package lmerTest, version 2.0–36^55^. Face stimulus coding for all models was as follows: women = − 0.5, men = 0.5, low status = − 0.5, and high status = 0.5. Perceiver gender was coded as follows: women = − 0.5, men = 0.5. We allowed for between-participants variance in intercepts (i.e., random intercepts) to account for participant-level variations in average neural response. For each ROI, we examined whether target gender, target status, perceiver gender, and all possible interactions predicted neural activity. We used Bonferroni correction to set our alpha level for the omnibus models (each of the five ROIs) to 0.01. To follow up on significant Target Status × Perceiver Gender interactions in each ROI, we tested the simple effects of status at each level of perceiver gender and the simple effects of perceiver gender at each level of status using dummy coding for target status and perceiver gender. These models accounted for variance associated with target gender by conserving the original contrast coding for target gender. For the follow-up models, we maintained a 0.05 alpha level.

#### PLS analysis

For this analysis, we used an existing MATLAB-based PLS analysis toolbox (https://www.rotman-baycrest.on.ca/index.php?section=84). In brief, PLS relates two blocks of data to one another. For our seed PLS analysis, one data block represented each participant’s fMRI contrast image for the contrast (high-SES faces > low-SES faces), and the other data block represented each participant’s average signal difference in the VMPFC ROI for high-SES faces minus low-SES faces. For our task PLS analysis, one data block represented whole-brain Blood Oxygenation Level-Dependent (BOLD) activity in the form of each participant’s beta maps for each condition versus the session mean, and the other block represented the study design (i.e., the four face conditions)^38^. The goal of these analyses was to find weighted patterns (i.e., latent variables—LVs) that best explain the covariance between blocks (i.e., “cross-block covariance”). These latent variables are computed via singular value decomposition. For further details on the specifics of PLS analysis, see previous work by McIntosh and colleagues^40^.

**Figure 2:**
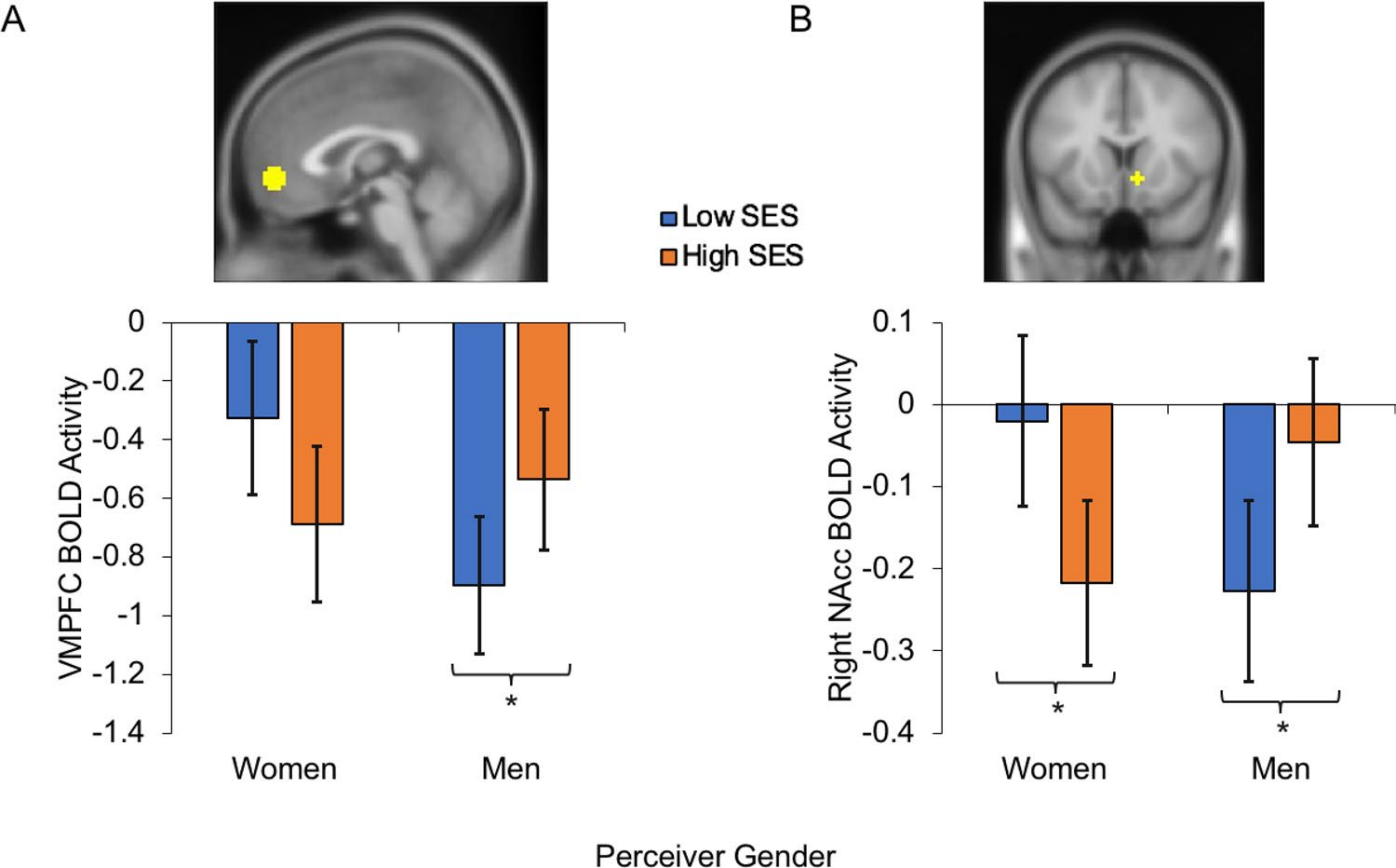
Average signal estimates in: (**A**) the ventromedial prefrontal cortex (VMPFC) and (**B**) the right nucleus accumbens (NAcc) as a function of perceived SES (low, high) and perceiver gender (women, men). Significant simple effects are indicated with asterisks, *p* < *.*05. Error bars indicate one SE unit above and below condition average. For full contrast statistics, see Table 1.

**Figure 3:**
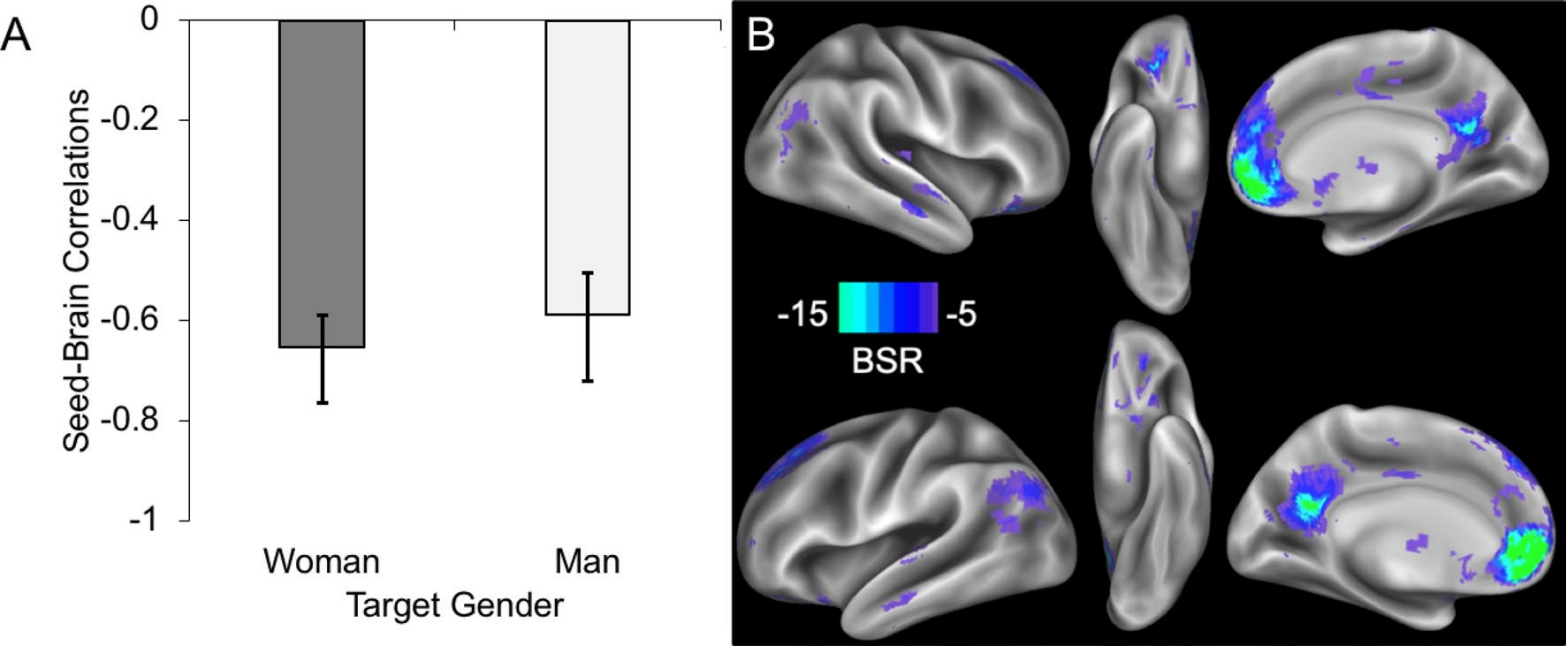
(**A**) Across both target genders, a significant latent variable emerged that captured a relationship between preferential responses to high (vs. low) SES in the VMPFC ROI and voxel co-activation in contrast images reflecting high SES > low SES. Seed-brain correlations plotted on the y-axis represent the strength of the relationship captured by the latent variable separately for faces depicting women and men. The error bars represent confidence intervals computed through a 2000-sample bootstrapping procedure^56^. (**B**) Voxel-wise co-activation patterns that most strongly contribute to the latent variable. Lateral, ventral, and medial views are displayed separately for the right hemisphere (top three images) and left hemisphere (bottom three images). Voxels were thresholded at BSR ≤ − 5. Note that the directionality of brain co-activation (**B**) needs to be interpreted in conjunction with the bar graph (**A**); lighter colored voxels indicate greater co-activation as a function of increasingly preferential VMPFC responses to high versus low SES.

The LVs reflect linear combinations of voxel activity throughout the brain that may be differentially instantiated for each experimental condition. To test the significance of each LV, we generated a set of 2,000 permuted samples using the same procedures and parameters reported in previous work^22^. The reliability of the original LV (i.e., its *p* value) is calculated as the proportion of the permuted singular values that exceed the singular value for the original LV. The reliability with which each condition contributes to the LV was determined using a bootstrap procedure. Specifically, we tested the reliability of condition-specific seed–brain correlations (for seed PLS) and brain co-activation scores (for task PLS) using 95% confidence intervals (see Figs. 2, 3). These confidence intervals were generated using a 2000-sample bootstrapping test. Because the top and bottom bounds of the confidence intervals are derived from a bootstrap distribution^22,56–58^, it is common for these bounds to be asymmetric relative to their corresponding estimates e.g.,^22,57,58^. The reliability with which each voxel contributes to the overall multivariate pattern captured by the LV (i.e., the voxel’s bootstrap ratio—BSR) is determined with a set of 2,000 bootstrapped samples^22^. For descriptive purposes, mapped BSR values were thresholded to approximate a 95% confidence interval, corresponding to BSR ≥ 3 or BSR ≤ − 3 for Task PLS. For the seed PLS analysis, we used a more stringent threshold of BSR ≤ − 5 in order to more clearly depict the resulting seed-based network, which is necessarily characterized by very large BSR values with increasing proximity to the seed coordinates. We used the standard cluster reporting function from xjView 97 (https://www.alivelearn.net/xjview), to extract and report clusters of 20 or more contiguous voxels containing BSRs that survived the above thresholds. This was not implemented for the purpose of statistical analysis, but rather to illustrate the peaks and extent of co-activating brain regions that characterize a significant latent variable.

In the present report, we used a seed PLS analysis to follow up on the univariate ROI analyses that showed main effects of perceived SES. Specifically, we examined the degree to which preferential responses to high-SES (vs. low-SES) faces in the VMPFC were associated with the extent of co-activation in participant-level whole-brain contrast images reflecting greater activity for high-SES (vs. low-SES) faces. We also used task PLS analysis to explore coordinated patterns of neural activity related to target gender and status across the whole brain without the constraint of any particular contrast (as in the seed PLS analysis). The task PLS analysis identified linear combination of voxels that optimally varied by gender and status of the faces. In our design, we had four viewing conditions: high-SES woman, high-SES man, low-SES woman, low-SES man. Finally, to explore effects of perceiver gender (which emerged in our ROI analyses—see “Results”), we also tested post hoc the magnitudes of each participant’s task PLS co-activation scores by condition as a function perceiver gender.

### Data and code availability

Raw data and analysis scripts are available for download (https://osf.io/pnqe7). These data were pre-processed and analyzed using SPM8 (www.fil.ion.ucl.ac.uk/spm) and a custom suite of scripts for fMRI analysis (https://github.com/ddwagner/SPM8w). PLS analyses were implemented using the PLS analysis toolbox (https://www.rotman-baycrest.on.ca/index.php?section=84). Average ROI signals extracted by SPM8w were analyzed using R (version 3.5.3).

## Results

### ROI analyses

#### VMPFC

For this region, we observed a significant Target Status × Perceiver Gender interaction (see Fig. 2A), *b* = 0.721, *SE* = 0.262, *CI*_*95%*_ = [0.207, 1.234], *t*(189) = 2.752, *p* = 0.007. All other effects were non-significant, *p* > 0.26. Analyses of simple effects (Table 1) revealed that only men exhibited significantly greater VMPFC activity in response to high-status (vs. low-status) targets. This pro-high-status bias occurred irrespective of target gender.

**Table 1:** Contrast statistics for target SES × perceiver gender interactions.

Region	Analyses	Group	*b*	*SE*	*CI*_*95%*_	*t*	*df*	*p*
VMPFC	High SES–Low SES	Men	0.361	0.172	[0.024, 0.698]	2.099	189	.037*
		Women	− 0.359	0.198	[− 0.727, 0.027]	− 1.821	189	.070
	Men–Women	High SES	0.152	0.307	[− 0.450, 0.754]	0.494	92.55	.622
		Low SES	− 0.569	0.307	[− 1.171, 0.033]	− 1.851	92.55	.067
Right NAcc	High SES–Low SES	Men	0.182	0.077	[0.032, 0.332]	2.374	189	.019*
		Women	− 0.198	0.088	[− 0.370, − 0.025]	− 2.242	189	.026*
	Men–Women	High SES	0.172	0.127	[− 0.076, 0.421]	1.359	99	.177
		Low SES	− 0.207	0.127	[− 0.456, 0.042]	− 1.632	99	.106

#### NAcc

In the right NAcc, there was a significant Target Status × Perceiver Gender interaction (see Fig. 2B), *b* = 0.379, *SE* = 0.117, *CI*_*95%*_ = [0.151, 0.608], *t*(189) = 3.25, *p* = 0.001. All other effects in the right NAcc and left NAcc were non-significant (*p* > 0.15). As in the VMPFC, analyses of simple effects (Table 1) showed that only men exhibited significantly greater right NAcc activity in response to high-status (vs. low-status) targets, irrespective of target gender.

#### Amygdala

The right amygdala showed a main effect of target gender indicating that perceiving faces of women (vs. men) elicited a greater response in this region, *b* = -0.194, *SE* = 0.077, *CI*_*95%*_ = [− 0.348, − 0.041], *t*(189) =  − 2.477, *p* = 0.014. The left amygdala showed a trend for an interaction effect between Target Gender × Perceiver Gender, *b* = 0.268, SE = 0.124, *CI*_*95%*_ = [0.026, 0.510], *t*(189) = 2.167, *p* = 0.032. However, the main effect of target gender and its interaction with perceiver gender did not reach significance at our Bonferroni-corrected alpha of *p* < 0.01. All other effects in the amygdalae were also non-significant, *p* > 0.07.

### Whole-brain regressions by perceiver gender

Complementing the ROI analyses, we tested for clusters across the brain that differed between men and women participants during impression formation for faces varying in gender and SES. Participant-level contrast images for the effects of target gender (woman > man), target SES (high SES > low SES), and the Target Gender × Target Status interaction (high-SES woman − high-SES man > low-SES woman − low-SES man) were analyzed at the group level using independent-samples *t*-tests to test for differences in these effects by perceiver gender. For these whole-brain exploratory analyses, we estimated a cluster extent threshold using AlphaSim. After 10,000 Monte Carlo simulations of all voxels at a voxel-wise false-positive rate of *p* < 0.001, we arrived at a cluster extent threshold of 52 voxels. It should be noted that cluster-level thresholds generated by AlphaSim do not adequately control for false positives in all conditions^59^. Therefore, these whole-brain analyses should be interpreted with caution, pending future replication.

#### Target gender effects

No significant clusters were observed.

#### Target SES effects

For the high SES > low SES target contrast, we observed five clusters where men relative to women exhibited more activity to high-SES than low-SES targets (see Table 2). Participants identifying as men exhibited greater activity for high than low SES in various areas of the prefrontal cortex; the ventral anterior cingulate cortex; the orbital frontal cortex; the cerebellum; and the middle occipital gyrus. There were no significant clusters showing more activity to high-SES than low-SES targets for participants identifying as women.

**Table 2:** Whole-brain univariate contrasts by participant gender.

Participant contrasts	Task contrasts (face effects)	Hemisphere	Region	*k*	*t*(1, 63)	*p*_*uncorr*_	Peak MNI Coordinates		
							x	y	z
Men > Women	High SES > Low SES	R	Premotor cortex	578	5.85	< .001	36	− 3	36
		R	Middle frontal gyrus		5.06	< .001	33	3	45
		R	Superior frontal gyrus		4.95	< .001	18	9	45
		L	Ventrolateral cerebellum	63	5.01	< .001	45	− 36	− 51
		L	Anterior cerebellum		3.87	< .001	36	− 33	− 42
		R	Ventral anterior cingulate cortex	120	4.89	< .001	15	24	− 9
		R	Orbitofrontal cortex		3.54	< .001	24	45	− 15
		L	Premotor cortex	333	4.75	< .001	− 24	0	42
		L	Superior frontal gyrus		4.60	< .001	− 15	18	36
		L	Middle frontal gyrus		4.49	< .001	− 30	9	39
		L	Middle occipital gyrus	67	4.56	< .001	− 36	− 84	18
	Men > Women		N/A						
	HSW–HSM > LSW–LSM		N/A						
Women > Men	High SES > Low SES		N/A						
	Men > Women		N/A						
	HSW–HSM > LSW–LSM		N/A						

*HSW* High SES Woman, *HSM* High SES Man, *LSW* Low SES Woman, *LSM* Low SES Man.

#### Target gender × target SES interaction

For the high-SES woman − high-SES man > low-SES woman − low-SES man interaction contrast, we observed no significant clusters for either men or women in our sample.

### PLS analyses

#### Seed PLS

Using seed PLS, we first explored the relationship between each participant’s preferential response to high SES in the VMPFC and co-activation across the brain in contrast images reflecting high SES > low SES. Results from this seed PLS analysis revealed a significant LV (*p* < 0.001) that captured a relationship between the average preferential VMPFC response to high (vs. low) SES (i.e., the seed) and co-activation of an extended network in the univariate high SES > low SES contrast images. This relationship was similar for faces depicting men and faces depicting women (see Fig. 3A). The regions that contributed most to this LV (see Fig. 3B and Table 3) included the ROIs that served as the focus for our univariate analyses as well as an extended set of regions including the precuneus, temporoparietal junction, insula, and orbitofrontal cortex. To further explore this functional connectivity network for each participant gender, we conducted the same seed PLS analysis separately by participant gender. In both cases, we obtained a significant LV featuring a very similar set of regions (see Supplemental Text S4). Indeed, the two LV maps showed a Pearson correlation of *r* = 0.51.

**Table 3:** Clusters identified by seed PLS (n = 65) with preferential VMPFC response to high SES as the seed.

Hemisphere	Region	Cluster size	MNI coordinates (mm)			BSR
			x	y	z	
**Decreased high > low contrast image co-activation with greater VMPFC preference for high (vs. low) SES**						
	N/A					
**Increased high > low contrast image co-activation with greater VMPFC preference for high (vs. low) SES**						
R	Cerebellum	1296	30	− 81	− 39	− 9.65
L	Cerebellum (anterior lobe)		− 21	− 50	− 31	− 6.87
L	Brainstem (pons)		− 12	− 35	− 35	− 5.24
L	Anterior inferior temporal gyrus	38	− 42	− 6	− 48	− 6.48
L	Cerebellum (posterior lobe)	378	− 54	− 54	− 27	− 7.34
L	Fusiform gyrus		− 51	− 60	− 23	− 6.65
L	Inferior temporal gyrus		− 60	− 51	− 16	− 5.84
R	Inferior temporal gyrus	431	60	− 57	− 21	− 7.90
R	Fusiform gyrus		51	− 61	− 25	− 7.15
R	Cerebellum (posterior lobe)		51	− 63	− 27	− 5.30
L	Middle temporal gyrus	341	− 66	− 12	− 12	− 8.93
L	Superior temporal gyrus		− 66	− 21	− 1	− 6.40
L	Insula		− 42	− 12	3	− 5.13
R	Superior temporal gyrus	378	60	− 3	− 12	− 10.06
R	Middle temporal gyrus		60	− 1	− 17	− 6.37
R	Temporal pole		60	4	− 18	− 6.16
R	Parahippocampal gyrus	74	24	− 24	− 21	− 9.10
	VMPFC	5311	3	54	− 3	− 60.34
	DPMFC		0	51	37	− 6.71
	ACC		0	42	16	− 8.14
L	Superior frontal gyrus		− 18	31	50	− 9.46
R	Superior frontal gyrus		21	36	46	− 9.41
	MOFC		0	41	− 19	− 8.91
L	OFC		− 24	41	− 15	− 6.83
R	OFC		27	38	− 20	− 5.60
R	Ventral striatum		9	15	− 10	− 7.07
L	Caudate		− 12	8	6	− 6.36
R	Caudate		9	5	3	− 7.46
L	Amygdala		− 21	0	− 15	− 7.71
L	Insula		− 33	16	− 14	− 6.46
R	Insula		36	14	− 14	− 5.24
	Thalamus		0	− 13	5	− 6.08
	Precuneus	1675	− 3	57	24	− 17.01
	Posterior cingulate		0	− 45	22	− 7.69
	Middle cingulate		0	− 18	41	− 7.15
	Vermis		0	− 64	− 1	− 6.40
	Cuneus		0	− 69	23	− 8.27
R	Temporoparietal junction (angular gyrus)	397	54	− 69	27	− 8.39
R	Middle temporal gyrus		54	− 69	9	− 6.12
L	Temporoparietal junction (supramarginal gyrus)	788	− 57	− 69	30	− 10.23
L	Temporoparietal junction (angular gyrus)		− 45	− 66	41	− 7.11
L	Middle temporal gyrus		− 57	− 69	20	− 8.51
R	Subgyral white matter (near insula)	23	33	− 3	24	− 5.94
R	Paracentral lobule	102	9	− 33	75	− 7.21

Cluster subregions are reported to illustrate the anatomical extent of the cluster beyond the peak BSR.

*R* right, *L* left, *BSR* bootstrap ratio—BSR indexes reliability of each cluster. All BSR ≤ -5; all clusters ≥ 20 voxels.

#### Task PLS

**Figure 4:**
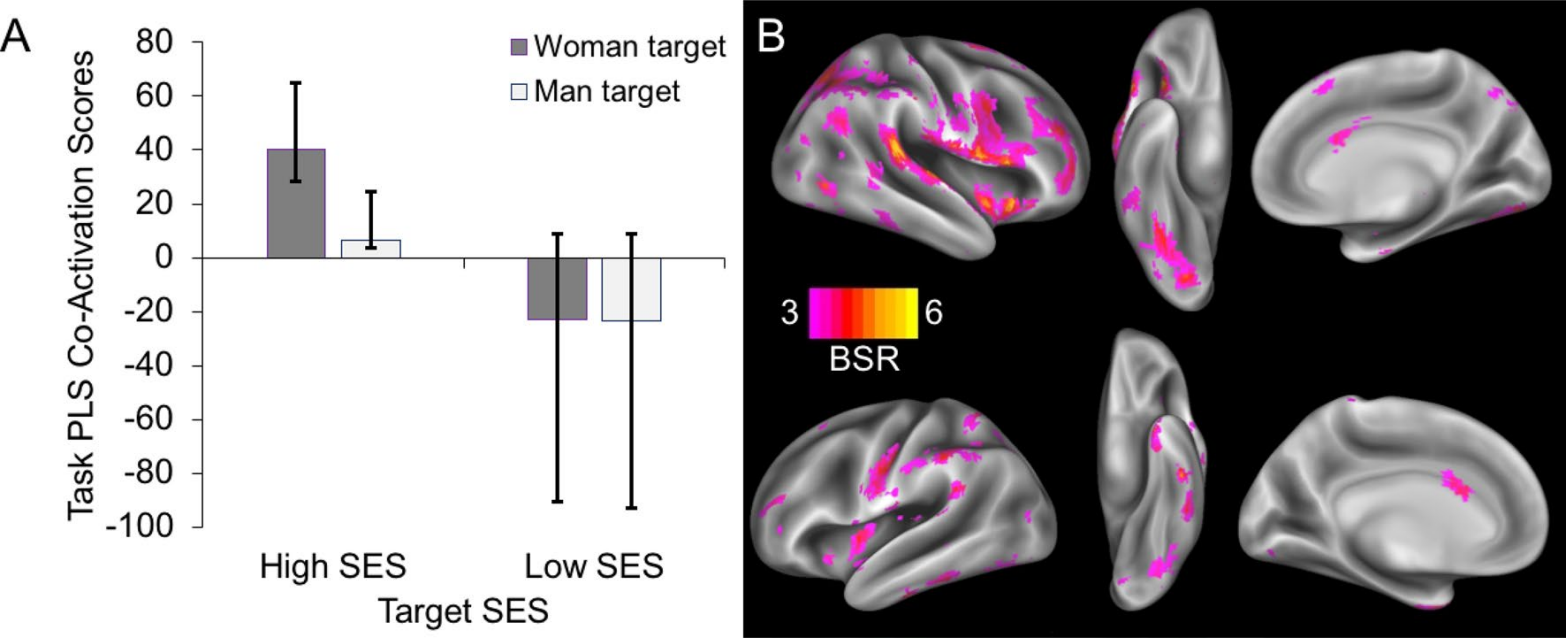
(**A**) The main effect of target status emerged as a significant LV in the task PLS. Task PLS co-activation scores (i.e., design salience scores, see y-axis) represent the strength of the relationship between the network of brain regions and the task conditions. The error bars represent confidence intervals computed through a 2000-sample bootstrapping procedure^56^. (**B**) Voxel-wise patterns that increased in co-activation for high-SES versus low-SES faces. Lateral, ventral, and medial views are displayed separately for the right hemisphere (top three images) and left hemisphere (bottom three images). Voxels were thresholded at BSR ≥ 3. Note that the directionality of brain activities needs to be interpreted in conjunction with the bar graph; warmer colors indicate greater co-activation when forming impressions of high-SES faces.

The task PLS analysis revealed one significant LV (*p* = 0.003), which explained 66.3% of the crossblock covariance compared to all other candidate LVs. We tested the extent to which the different experimental factors contributed to this significant LV by conducting a 2 (Target SES: low, high) × 2 (Target Gender: woman, man) repeated-measures ANOVA (Type III) on the participant-level task PLS design salience, plotted in Fig. 4A. Results revealed this LV was best characterized by a significant main effect of ascribed SES, *F*(1, 64) = 12.429, *p* = 0.001, *CI*_*95%*_ = [19.657, 73.719]. All other effects were non-significant, *p* > 0.24. We next plotted all voxels contributing most strongly to this LV (see Fig. 4B). Specifically, we observed increased neural co-activation when participants formed impressions of high-SES (vs. low-SES) faces in a broadly distributed network consisting of frontoparietal and insular brain regions, inferior/superior temporal regions, cingulate cortex, and cerebellum, together with extensive co-activation in regions like the pons and midbrain (see Table 4). The regions constituting this network largely coincided with those involved in salience and attention^60–62^, possibly encompassed by more extended domain-general networks supporting focused task engagement^63–65^. We revisit this network in greater detail in the Discussion.

**Table 4:** Task PLS (n = 65) reveals a network that co-activates more for high-SES (vs. Low-SES) targets.

Hemisphere	Region	Cluster size	MNI coordinates (mm)			BSR
			x	y	z	
**Increased co-activation with lower status**						
	N/A					
**Increased co-activation with higher status**						
L	Cerebellum	2591	− 39	− 57	− 42	4.71
R	Cerebellum		45	− 65	− 24	4.61
R	Lingual gyrus		21	− 78	− 9	4.21
L	Cerebellum	75	− 30	− 33	− 51	5.18
R	Cerebellum	21	30	− 30	− 48	5.25
L	Temporal pole	111	− 24	6	− 39	3.91
	Pons	25	0	− 27	− 30	3.61
R	Temporal pole	24	33	3	− 33	3.84
L	Inferior temporal gyrus	123	− 60	− 45	− 21	4.37
L	Fusiform gyrus		− 52	− 51	− 21	3.44
R	Posterior superior temporal gyrus	2858	66	− 30	15	5.34
R	Supramarginal gyrus		63	− 24	18	4.92
R	Insula		36	18	− 9	4.46
R	Middle frontal gyrus		52	50	7	4.32
R	Superior parietal lobule		21	− 60	69	4.06
R	Postcentral gyrus		60	− 22	50	3.95
R	Inferior frontal gyrus		51	9	39	3.81
R	Midbrain	63	33	− 27	3	3.95
L	Insula	41	− 39	− 3	6	4.04
R	Middle occipital gyrus	21	42	− 84	6	3.60
L	Middle frontal gyrus	74	− 39	45	18	3.68
L	Superior frontal gyrus		− 31	57	18	3.49
L	Supramarginal gyrus	49	− 57	− 45	24	3.96
L	Precentral gyrus	584	− 48	− 12	30	4.39
L	Supramarginal gyrus		− 51	− 45	60	4.09
L	Postcentral gyrus		− 48	− 18	33	4.06
L	Inferior parietal lobule		− 48	− 42	45	3.95
R	Middle cingulate gyrus	71	6	3	33	3.80
L	Precuneus	25	− 21	− 66	36	3.39
R	Middle frontal gyrus	205	27	3	66	4.37
R	Superior frontal gyrus		21	30	57	3.44
L	Middle frontal gyrus	48	− 24	24	60	4.06
R	Superior frontal gyrus	41	9	− 6	84	3.62

*R* right, *L* left, *BSR* bootstrap ratio—BSR indexes reliability of each cluster. All BSR ≥ 3; all clusters ≥ 20 voxels.

Cluster subregions are reported to illustrate the anatomical extent of the cluster beyond the peak BSR.

**Figure 5:**
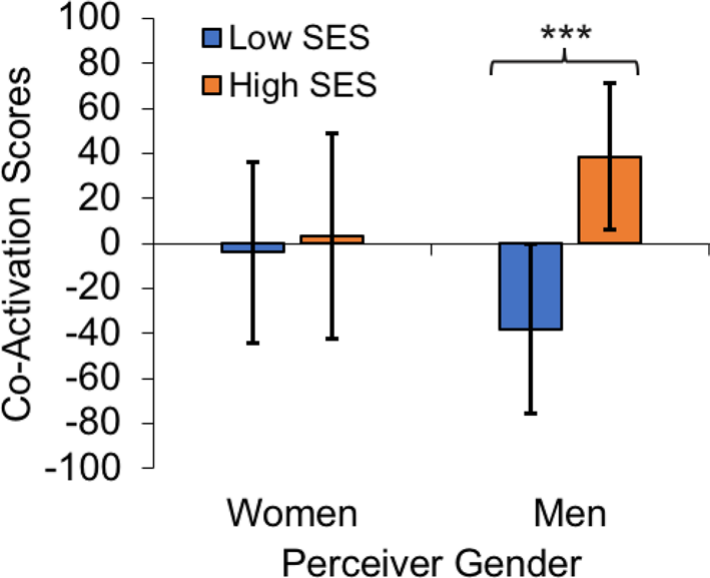
Men showed a larger effect of perceived SES on brain network co-activation. Participant-level design salience score estimates from task PLS that were averaged to create Fig. 4A were extracted and analyzed by participant gender. Results revealed that network sensitivity to target SES was driven primarily by men in the sample. Men showed a significant effect of SES in their brain co-activation scores, as indicated by the asterisks, *p* < .001. Error bars indicate a 95% confidence interval in *t*-tests against zero, which represents the overall mean contribution to the latent variable across all task conditions and participants. In contrast with the seed and primary task PLS analyses (Figs. 3 and 4, respectively) which utilized bootstrapping to generate confidence intervals, here we generated confidence intervals from between-participants variance in condition-specific co-activation estimates for each participant, which were the units for this analysis.

Follow-up analyses revealed that this network was driven primarily by participants identifying as men (Fig. 5).

Following up on effects of perceiver gender in the ROI analyses, we explored whether the status-sensitive network of brain co-activation captured by the LV may be modulated by perceiver gender. To do this, we extracted participant-level design salience scores from the task PLS analysis and submitted those scores to a 2 (Target SES: low, high) × 2 (Target Gender: woman, man) × 2 (Perceiver Gender: woman, man) mixed ANOVA (Type III), with perceiver gender as a between-participants factor. Results again revealed a significant main effect of target SES, *F*(1, 63) = 10.79, *p* = 0.002, *CI*_*95%*_ = [19.657, 73.719]. Importantly, this effect of target SES was significantly modulated by perceiver gender, *F*(1, 63) = 7.44, *p* = 0.008 (Fig. 5). All other effects were non-significant, *p* > 0.25. Analyses of simple effects revealed that the effect of target SES on participant-level design salience (i.e., brain co-activation) scores was reliable for men in the sample, *t*(36) = 4.137, *p* < 0.001, *CI*_*95%*_ = [39.066, 114.210], but not for women, *t*(27) = 0.44236, *p* = 0.66, *CI*_*95%*_ = [− 25.873, 40.096]. Effects of participant gender on participant-level design salience scores for each target SES level were not significant, *p* > 0.20.

### Summary of stimulus ratings and responses

To provide greater context on the faces on which participants formed their impressions, we assessed likability ratings, feeling thermometers, and SES recall performance for all face stimuli presented during the two scan runs. Analyses predicted likeability, warmth, or recall as a function of the participant’s gender and the four task conditions. We summarize the results from those tasks here, but full analyses can be found in Supplemental Text S4. For the likeability ratings and feeling thermometers, participants rated faces of women more positively than faces of men. This effect was significant for men and women participants but was especially pronounced among women for the feeling thermometers (i.e., perceived warmth). We found no other significant effects involving ascribed face SES nor participant gender. The status recall results showed above-chance recognition of ascribed SES levels for the face stimuli (65.15% across all participants), despite the lack of instructions to memorize face-status pairings. SES recall was not significantly impacted by face SES, gender, or participant gender. Finally, we tested the possibility that men and women were forming impressions of target faces at different speeds. RT data were analyzed using a mixed linear model that included fixed effects for target gender, target SES, participant gender, and all possible interactions between these terms (and random effects by participant for the main effects of target gender and SES and the interaction between these terms). Results indicated that all effects from this model were non-significant, *p* > 0.06. Additional simple comparisons confirmed that women and men showed no significant differences in impression formation response times for any of the four face conditions, *p* > 0.11.

Taken together, our contextual analyses of the self-report data showed some limited evidence that participants’ explicit evaluative ratings were sensitive to both perceived SES and gender, albeit only for recall and likeability/warmth ratings, respectively. Perceived SES did not bias any explicit post-scan ratings of the face stimuli. Although the likeability/warmth ratings did not parallel the fMRI findings, this could be explained by key differences between the tasks. Whereas impression formation is more holistic and likely integrates a number of dimensions beyond likeability/warmth (e.g., competence), likeability and warmth judgments are more narrowly focused. Additionally, as indicated in our rationale for choosing this paradigm, the impression formation task completed in the scanner was more private in the sense that participants’ responses were not indicative of their impressions, unlike in the explicit ratings tasks. Accordingly, likeability and warmth ratings are more susceptible to socially desirable responding.

## Discussion

Across complementary univariate and multivariate analyses, the present findings reveal consistent evidence of greater sensitivity to status (viz., SES) in men than in women. In line with previous findings^6,28^, we observed greater activity in brain regions indexing positive evaluations of others^26^ and social reward/salience (e.g., NAcc, amygdala) as men (but not women) formed impressions of high-SES (vs. low-SES) faces. Seed PLS analyses revealed that preferential responses to high (vs. low) SES in the VMPFC were associated with greater co-activation in contrast images reflecting high SES > low SES within an extended network involved in person evaluation and mentalizing, including the precuneus, temporoparietal junction, insula, and orbitofrontal cortex. This co-activation pattern was similar for women and men, suggesting that despite a greater VMPFC preference for high SES in men, both genders show similar patterns of functional connectivity when they do show a pro-high-SES bias in the VMPFC. Beyond our focal regions of interest involved in person evaluation, an exploratory task PLS analysis revealed a coordinated network of brain regions that was sensitive to high-SES (vs. low-SES) faces. Again, this pattern was only reliably observed for men in our sample. The functional network emerging from these analyses is consistent with greater relevance and/or engagement with status cues in men. Taken together, the findings provide evidence that high versus low SES is associated with neural responses indicative of positivity, reward, and salience during impression formation for men. The present findings are noteworthy for their contribution to a network neuroscience understanding of status perception but also for their implications regarding how gender and status interact to shape our impressions of others. We elaborate on these implications in separate sections below. Additionally, by examining both target and perceiver gender, this work provides greater representation in samples than is often used for psychophysiological studies and avoids the potential pitfall of assuming that women respond to status in the same way that men do^66^. Indeed, as the present and previous work suggest, this is only sometimes the case.

### Toward a network neuroscience approach to social hierarchy perception

The present study is among the first to use a multivariate brain network approach to investigate status-based impression formation. This data-driven approach revealed a functional brain network responsive to perceiving high-SES compared to low-SES people, particularly in men. This network shows considerable overlap with a previously identified set of regions involved in status-based attention^6,67^ and more broadly with networks believed to support salience detection^60,62^ and attention^61,62^. However, the observed co-activation network extended beyond these core networks, possibly implicating broader domain-general networks supporting focused task engagement^62–65^. Complementing our ROI approach, which focused on brain regions previously shown to support status-based evaluations^6,28^, the preferential involvement of this large brain network was also driven primarily by men in our sample (see Fig. 5). Taken together, these findings suggest that men may be especially engaged when forming impressions of high-SES people and that these impressions are likely positive^14^.

It is worth noting that the inferior parietal cortex emerged as one component of the status-based attention network identified through task PLS analysis. This region has been implicated in computing social distances^68–72^, showing greater activity for difficult comparisons between two closely ranked individuals compared to easy comparisons between very differently ranked individuals^71^. The involvement of the inferior parietal cortex in the SES-based functional network suggests that men in our sample may dedicate greater attention to differentiating SES when forming impressions of others, particularly when those individuals are high in SES. This interpretation would be consistent with findings from the human and animal literature that high-status (vs. low-status) individuals more readily attract and/or re-direct attention^6,73–76^ and are more easily remembered^76^.

### Implications for status and gender of the perceiver

One key takeaway in the present study is that the participant’s gender consistently altered sensitivity to perceived SES. In multiple regions thought to support status-based evaluations^6,25,28^, men more reliably showed neural responses that were more indicative of positive impressions for high- versus low-status targets. Men also showed greater coordination for increasing target SES in an extended set of regions implicated in salience, attention, and task engagement as discussed in the preceding section. In other words, men more than women showed evidence of positive evaluations and more deliberative engagement with high-SES faces during impression formation. This finding is consistent with evolutionary psychological accounts suggesting that men more readily display, attend to, and/or pursue higher status^18,77,78^.

Notably, the effect of perceiver gender on neural responses to target SES was not further modulated by target gender. Based in part on predictions derived from theories of sexual selection^19^, it has been argued that women and men may prefer different dimensions of status, both for themselves^77,78^ and for potential heterosexual mates^19,20,79^. Despite some support for this theory in the behavioral literature, it receives little support from the extant albeit sparse neuroimaging literature on gender and status^36^. One important caveat is that no fMRI study to date has explicitly invoked a context relevant to mating.

### Implications for status and gender in person perception

#### Operationalizing target status

Status can be conveyed along multiple dimensions ranging from commonly studied attributes like dominance^36^ and finances^23,24,80^ to social categories such as race^21,22,81^ and gender^36^. One important aspect of this study is its focus on SES instead of dominance, which has perhaps received greater attention so far in neuroimaging studies of gender in hierarchical contexts^36^. As a measure of social rank, SES comprises an individual’s standing in terms of education, income, and occupational prestige^82^. Although SES may convey dominance in some contexts, these two constructs are not the same^6,83^. Accordingly, neural responses to perceived dominance may not reflect responses to status when it is operationalized in terms of SES. Indeed, previous work suggests that VMPFC responses to high status depend on the dimension of status in question, with greater responses for high moral status than for high financial status^23,24^.

In addition to the dimension of status in question, it is also important to consider the means by which status is conveyed. In the present study, status was conveyed through colored cues previously paired with high or low SES. This approach is particularly important for neuroimaging studies of status due to potential confounds that exist in more naturalistic and subtle cues of status such as facial cues^84^ and clothing^81,85–87^. For example, clothing can shape impressions of dominance^85^, competence^86,87^, and attractiveness^88^, all of which are imperfectly tied to perceptions of status^83^. This is important for neuroimaging studies of status for two reasons. First, using such cues makes it difficult to distinguish whether purported effects of social rank may instead be due to related but non-equivalent attributes such as competence. Second, this problem would be compounded when comparing the perception of two groups that stereotypically differ in terms of competence^87^. The present study largely bypasses these limitations by relying on ascribed SES knowledge through color assignment rather than clothing or facial expressions.

#### Absence of effects of target gender

Although the gender of perceivers shaped neural responses to target status during impression formation, these responses did not differ based on the gender of targets. This is noteworthy for a few reasons. First, participants did explicitly rate women are more likeable than men after scanning, suggesting that they did attend to gender to some extent. Additionally, previous work has revealed distinct neural correlates of target gender, regardless of whether gender is explicitly processed^36,89^. Given the relative salience of social category cues such as gender^90,91^, we anticipated that gender might interact with target status. Indeed, Marsh and colleagues^36^ observed greater responses in the VMPFC and right amygdala for increasingly dominant body postures of only the woman targets. However, as previously mentioned, the fact that status was based on different dimensions (SES vs. dominance) and antecedents (perceptual attributes vs. person knowledge) may explain these differences. Furthermore, neuroimaging work examining status-based impression formation in the presence of another salient social category (viz., race) also only found effects of status^21,22^. One possibility is that participants disregarded initial impressions based on gender information by focusing on the available status knowledge to form more individuated impressions^92^.

The absence of effects of target gender is also noteworthy from the perspective of some proposals arising from evolutionary psychology. Due in part to a lower minimal male investment in child-rearing^93^, evolutionary psychologists argue that males were frequently engaged in ancestral conflicts within and between groups, resulting in greater natural selection of physical and psychological characteristics associated with dominance in males than in females^18^. This relationship between sex and social hierarchy cues may also extend to social constructions such as the clothes we wear. For example, previous work has shown that men wearing high-status attire are more readily attended^94^ and rated as more competent^87^ than women wearing high-status attire. In contrast with this previous work, the present study showed no unique effects of SES for faces depicting men compared to women. One possibility is that conveying status through person knowledge rather than appearance eliminates potential gender bias in how status shapes social attention and evaluations^6^.

### Alternative interpretation

Prior to concluding, it is worth considering an alternative interpretation of the present work. Given that the impression formation task did not include a control task condition, one could argue that our findings are not related to the status information ascribed to each target during impression formation. In this perspective, one could imagine obtaining similar findings if the faces of women and men were grouped based on other social information than social status such as their preference for different kinds of food. If so, then our findings might mean that men are more sensitive than women to social information in general rather than social status, per se.

Notwithstanding existing research suggesting that, in some instances, women may be more sensitive to social information than men^95–97^, we believe it is more parsimonious to focus our interpretation in the context of status sensitivity, rather than sensitivity to social information more generally, for three reasons. (1) Given that the face stimuli were equated across various dimensions and counterbalanced across participants within gender, we think it would be prudent not to over-generalize our findings to other social information. (2) The present findings are consistent with the literature suggesting greater status-driven perception and behaviors in men^10,14–18,98^. (3) Assuming that men were more sensitive to social information in general, it is unclear why men didn’t also show greater neural sensitivity to perceived gender, a dimension that has demonstrable biological and psychological relevance as a function of perceiver gender^19,20^. The present study was focused primarily on SES as the perceived status dimension, finding evidence of greater neural sensitivity to high (vs. low) SES in men compared to women. Importantly, SES is but one possible dimension of social status, being composed of subcomponents (e.g., income, education, occupational prestige) that may or may not elicit the same kinds of evaluations^6,28,83^. Other social hierarchies that have been studied using fMRI include hierarchies based on dominance/ability/competence^31,99^, power^80,100^, and moral character^23,24^. With few exceptions^23,24^, these studies have focused on just one hierarchy dimension and often just one gender, usually men. In general, these studies frequently find the VMPFC and NAcc are responsive to high-ranking individuals. Other work by Kumaran and colleagues suggests that the VMPFC in particular may be implicated in updating social hierarchy knowledge in hierarchies involving the self^100^. However, it is unclear whether these effects are similarly driven by men as they were for SES in the present study. In future research, it will be important to replicate and extend the present work to determine whether men also show greater neural sensitivity to high status compared to women for other status dimensions as well as for social dimensions that are potentially ordinal but not frequently used to delineate hierarchies (e.g., age).

In sum, we believe our present findings can be interpreted as a gender-specific differential in sensitivity to perceived status. However, we do not believe that men’s greater sensitivity to SES in this study precludes the possibility that women (or men) might be more responsive to other social information in other contexts. For example, it is possible that subsequent studies may uncover a preference in women for other dimensions of status such as communal/moral character^23,24,77^ or that women may value high-SES individuals more than men in certain contexts. These questions among others are ripe for future inquiry and consistent with recent calls to explore how perceiver gender may differentially shape sensitivity to other dimensions of social information^66^. We hope that this study will pave the way for future work exploring gender differences in sensitivity to different forms of social information.

## Conclusion

The social psychological literature is full of examples illustrating the barriers faced by women who seek to ascend the social hierarchy^88,101^. The present findings help to identify potential neurobiological constraints that may be accounted for in interventions aimed at reducing the barriers to and promoting women’s participation in leadership^101^. Specifically, this study provides evidence that men may place greater emphasis on SES than do women when they privately form impressions of others. Specifically, men showed greater responses to high versus low SES in brain regions shown to index positive status-based evaluations, such as the VMPFC and NAcc^6,25,28^. Moreover, men showed greater coordinated activity toward high-SES faces in a functional network comprising brain regions implicated in salience, attention, and/or task engagement. These findings are consistent with proposals that men are more sensitive to status than women^10,17,18,77,102^. However, as with any research uncovering gender differences, it is important to interpret the present findings with caution^103^. Just because women showed less sensitivity than men to ascribed SES in the present context does not mean that women are generally insensitive to social status during impression formation. Indeed, similar seed PLS results for men and women in the present study suggest that both genders may use a similar functional connectivity network for evaluating SES during impression formation even if they may differ in their average VMPFC responses to SES.

### Diversity statement

Recent work in psychology and neuroscience has identified a bias in citation practices such that papers from women and other minorities are under-cited relative to the number of such papers in the field^104,105^. Here we sought to proactively consider choosing references that reflect the diversity of the field in thought, form of contribution, gender, and other factors. We obtained predicted gender of the first and last author of each reference by using databases that store the probability of a name being carried by a woman^105,106^. By this measure (and excluding self-citations to the first and last authors of our current paper), our references contain 23.3% woman(first)/woman(last), 12.2% man/woman, 14.4% woman/man, 50% man/man, and 0% unknown categorization. This method is limited in that a) names, pronouns, and social media profiles used to construct the databases may not, in every case, be indicative of gender identity and b) it cannot account for intersex, non-binary, or transgender people. We look forward to future work that could help us to better understand how to support equitable practices in science.

## Supplementary information

Supplementary Information

## Data Availability

Raw data and analysis scripts are available for download from https://osf.io/pnqe7.
