# Supplementary material for: Regional and network neural activity reflect men’s preference for greater socioeconomic status during impression formation
Source: Sci Rep. 2020 Nov 20;10:20302. doi: 10.1038/s41598-020-76847-z (PMC7679381; doi:10.1038/s41598-020-76847-z)
Supplement: Supplementary file 1 — Supplementary Information [file 41598_2020_76847_MOESM1_ESM.docx]

SUPPLEMENTAL MATERIAL

**Regional and network neural activity reflect men’s preference for greater socioeconomic status during impression formation**

Denise M. Barth^1*^, Bradley D. Mattan^2*^, Tzipporah P. Dang^1^, & Jasmin Cloutier^1^

* Shared First Authorship

Affiliations:

^1^ Department of Psychological and Brain Sciences, University of Delaware, Newark, DE, 19716 USA

^2^ Annenberg School for Communication, University of Pennsylvania, Philadelphia, PA, 19104, USA

Corresponding author:

Jasmin Cloutier

Department of Psychological and Brain Sciences

University of Delaware

105 The Green

Newark, DE 19716

Telephone: +1 (302) 831-4813

Email: jasmin.cloutier@gmail.com

# S1: Supplemental Measures and Screening Parameters

Individual differences were assessed in this experiment for participant screening, exploratory analyses, and future analyses of anatomical and resting-state data that are unrelated to the functional scans that were the focus of analyses in the main text. We summarize measures at five different points in the study: (1) initial screening, (2) online pre-testing, (3) surveys completed on the day of the scanning session but prior to entering the scanner, and (4) surveys and tasks completed immediately after scanning. All measures are available upon request.

## Participant Screening

People inquired about the study via e-mail and were sent an initial online screening survey to fill in demographics and health information: Race, ethnicity (Hispanic/non-Hispanic), gender, age, native language, dominant hand, U.S. residency for past five years, U.S. citizenship, country of birth, state/providence of birth; history of head injury, color blindness, psychotropic medications, drug use, developmental disorder diagnosis, chronic health condition, dental or non-removable metallic implants. Survey participants also provided contact information (viz., phone number and/or e-mail address). A research assistant contacted people who met the following criteria: (1) self-identify as White (non-Hispanic), (2) between 18 and 35 years old, (3) have lived in the U.S. for at least 5 years, (4) have a good command of the English language, (5) no history of drug abuse, (6) right handed, (7) no history of serious head injury, (8) no color vision problems, (9) no current acute illness, (10) not currently taking psychotropic medication, (11) no diagnosis of developmental disorders, (12) no diagnosis of a chronic disease that compromises mental, neural, or autonomic function, and (13) pass a standard MRI safety screen. A research assistant conducted phone interviews to fill out an MRI safety screening form and schedule the MRI appointment.

## Online Pre-Testing

We detail here any measures relevant to gender and/or status that were included in the online pre-testing survey. Measures that are not germane to the present study’s focus on gender and status will be analyzed for separate projects.

Of the 69 participants recruited, 24 participants were recruited from two prior neuroimaging experiments conducted by our research group. To avoid the redundant administration of measures that were previously collected, these 24 participants were given an abridged version of the pre-test survey that contained only novel measures. Relevant measures completed by participants from the first prior study^1^ included intergroup anxiety for high- and low-status men and women, objective SES, and demographics questions. Relevant measures completed by participants from the second prior study included the social dominance orientation scale, intergroup anxiety for high- and low-status men and women, feelings thermometers for high- and low-status men and women, MacArthur scale of subjective status, objective SES, and questions about parents’ incomes and jobs.

**Objective SES.** Objective socioeconomic status (SES) of the participant was assessed using a battery of measures assessing participants’ education, income, and assets^2^.

**Subjective SES.** Subjective social status was assessed using a single-item measure assessing family social class and the MacArthur Scale of Subjective Social Status^3^. Participants responded using a 10-point scale, from “1^st^ step” (lowest labeled step) to “10^th^ step” (highest labeled step). A second version of the MacArthur scale asked participants to instead consider their general standing in the context of their local community but without explicit reference to SES-related information.

**Social dominance orientation.** This 16-item measure assesses the endorsement of beliefs regarding the legitimacy of inequalities between social groups^4^. Responses were provided on a seven-point scale ranging from one (strongly disagree) to seven (strongly agree).

**Intergroup anxiety.** This 17-item measure asks participants to rate their thoughts or feelings regarding an anticipated collaborative interaction (real or hypothetical) with someone from a different social group^5^. Participants completed this measure six times, once for each of six hypothetical collaborators: high-status female, low-status female, high-status male, low-status male, Black individual, and White individual. The order in which the collaborators were presented was randomized for each participant. Responses were provided on a seven-point scale ranging from one (strongly disagree) to seven (strongly agree).

**Feeling thermometers.** Participants completed feeling thermometers separately for: high-status women, low-status women, high-status men, low-status men, Black people, and White people. For each group, participants responded to the following prompt using a sliding scale from 0 to 100: “Please rate how cold or warm you feel towards the group below (0 = coldest feelings, 50 = neutral, 100 = warmest feelings).”

**Social-cognitive measures for resting-state and anatomical analyses.** Unrelated to the present investigation, we also assessed several other social-cognitive measures for large-scale analyses of resting state and structural imaging data. Because these measures are unrelated to the fMRI experiment on gender and status, we do not report these measures here. However, a full list of these measures is available upon request.

**Miscellaneous demographics.** At the end of the pre-testing session, participants completed a battery of demographic items. Participants responded to questions regarding age, sex, race/ethnicity, political affiliation, conservatism/liberalism, high school type, native language, citizenship, country of origin, and religious affiliation. Some measures of subjective SES (e.g., family social class) and objective SES (e.g., education level and occupational prestige, childhood postal codes, K–12 educational institutions, parental education, income and occupational prestige) were also included at the end of this section. Items were each rated on a seven-point scale from one (not at all) to seven (very strongly/important/often).

One participant recruited for this experiment failed to complete the demographics portion of their pre-test survey. Furthermore, some participants initially recruited for previous studies did not give their age (*n* = 23) because we allowed them to skip survey questions they previously answered. To accurately assess age, we manually added one year for every year (up to two) that had passed since the date of their initial age assessment.

## Pre-Scan Measures

Participants filled out a brief survey prior to being prepared for entering the scanner with the following measures.

**State-trait anxiety inventory.** The State-Trait Anxiety Inventory (STAI)^6^ measures state and trait anxiety levels. Participants reported the intensity of their anxiety. Items for trait anxiety were rated on a four-point scale from one (almost never) to four (almost always); items for state anxiety were rated on a four-point scale from one (not at all) to four (very much so).

**Single-item stress/anxiety scales.** Participants responded to three questions about their current anxiety level, current stress level, and their stress level over the past month. Each item was rated on a sliding scale from one (least stressed) to 100 (most stressed).

**PANAS.** Participants completed the first Positive and Negative Affect Schedule (PANAS)^7^. In this study, participants rated their experience of each of the 20 affective states over the “past few hours” on a five-point scale from one (very slightly or not at all) to five (extremely).

## Post-Scan Measures

**Police–Civilian Videos.** At the completion of all scanning (described in the main text), our participants were taken back to the study testing room. As part of an unrelated exploratory analysis they watched 38 video clips (19 Black civilians and 19 White civilians) that were previously equated by civilian race on various dimensions (e.g., aggression, arousal) and gave explicit ratings of each clip on a scale from one (Not at all) to seven (Extremely). Participants responded to four questions for each clip: “To you, how aggressive does the civilian appear to be?” “To you, how guilty does the civilian appear to be?” “To you, how aggressive does the police officer appear to be?” “To you, how legitimate does the police officer’s aggression level appear to be?” Half of the participants rated the civilians first (civilian aggression, civilian guilt, officer aggression, officer legitimacy) and the other half rated the officers first (officer aggression, officer legitimacy, civilian aggression, civilian guilt).

**Contact with Police.** Participants responded to an adapted six-item scale regarding contact and quality of contact with police officers^8^.

**Evaluative Priming Task.** Following previous work^9^, participants were first familiarized with the priming task by categorizing ten valenced words (i.e., PARADISE, POISON) as positive or negative using the appropriate keys. We counterbalanced the key-mapping for categorizations. There was only one correct response per word. For the actual evaluative priming task, participants were taught to continue to categorize valenced words, one at a time, but now a face from the impression formation task (described in the main text), with the same color border, preceded each word. The first eight trials of the evaluative priming task were practice trials using different faces than from the impression formation task. The target stimuli used for practice and task familiarizing were different from those utilized in the experimental task.

The remaining 168 experimental trials followed seamlessly from the practice trials. Each possible type of trial (i.e., eight possible prime and target pairs) was repeated 21 times. Each face prime was shown six times throughout the task with the same status and color seen in previous tasks. The trials were randomly presented without replacement according to the trial type—they only repeated once all 28 distinct face primes showed once. Each face prime showed for 300 ms, and then was replaced by a target word for 1500 ms or until response. Failures to respond after 1500 ms resulted in termination of the trial and were recorded as an incorrect response. An intertrial interval (ITI) of 500 ms followed the response, preceding the next trial.

**Likeability rating task.** Participants completed a measure of explicit likeability for each of the 28 face stimuli presented during the impression formation task in the scanner. Faces were presented with the same status-associated colored backgrounds used in the impression formation task. Participants rated each face on a scale from one (extremely unlikeable) to nine (extremely likeable).

**Status recall.** Participants viewed all 28 faces from the likeability task, but this time without any status-associated color backgrounds. For each face, participants then indicated to the best of their ability whether the face was low or high in status.

**Trust game.** We adapted the trust game to the context of the present experiment^10^. At the start of the trust game, participants learned that the faces presented in the task were participants from a previous study whose responses to real financial trust decisions were recorded. Participants also learned that the outcome of one of their financial trust decisions would be randomly selected to compute a bonus payout to one of the participant’s interaction partners (i.e., a participant in a previous study). As in previous work^10^, participants began each trial with a 10 dollar allocation. Participants completed 28 trust game trials, one trial for each of the 28 identities presented to participants in the scanner. Faces of women and men were presented together with their status-associated background colors (see main text for details). Data from this task were intended to be part of a separate project on decision making, and so we are unable to report results from the trust game at this time.

**PANAS.** Participants completed the PANAS again^7^. As they did prior to scanning, participants rated their experience of each of the 20 affective states over the “past few hours” on a five-point scale from one (very slightly or not at all) to five (extremely).

**Gender and status contact.** The first section of this multi-item measure, adapted from Cloutier, Li, and Correll^11^, asks participants to report the SES and gender diversity of people they knew at three distinct stages of their childhood (e.g., ages 0–6, 6–12, and 12–18) and currently. We measured contact with high-status women, low-status women, high-status men, and low-status men throughout the lifespan^12^. Items asked about the percentage of friends, acquaintances, daily contacts, and media figures that are high/medium/low SES and the percentage of these categories who were female and male. The measure also assesses participants’ awareness of their environment’s gender and SES diversity and disparities.

**Modern sexism.** Participants responded to an eight-item measure indicating endorsement of statements regarding present-day sexism and its prevalence^13^. This measure used a five-point scale from one (strongly agree) to five (strongly disagree).

**Ambivalent sexism.** Participants completed a 22-item measure indicating endorsement of ambivalent sexist beliefs^14^. The items include statements about benevolent and hostile sexism and used a six-point scale from one (disagree strongly) to six (agree strongly).

**Feminist identification.** Participants were asked to respond “Yes” or “No” to the question, “Do you consider yourself a feminist?”

# S2: Supplemental Procedures

The study reported in the main text was run in the context of a protocol supporting multiple studies. Key aspects of the protocol pertaining to the present study of gender and status during impression formation are included in the main text. However, for completeness, we provide a supplemental overview of aspects of the protocol that were not germane to the focus of the study presented in the main text. A majority of the computerized tasks (including the impression-formation and cop–civilian fMRI tasks and their respective training procedures, likeability ratings, status recall, and the trust game) were presented using E-Prime 3.0 Professional^15^ except for the gender-status evaluative priming task which was presented in Inquisit^16^. As in the online pre-testing, all surveys were presented on the Qualtrics platform^17^.

## Pre-Scan Procedure

After the participant signed all consent forms and imaging center paperwork, participants completed a brief online survey consisting of the following measures: State and Trait Anxiety, single-item stress/anxiety scales, and PANAS (see Supplemental Text S1 for details).

## Scanning Session

After situating the participant in the scanner, we ran a reference scan to survey the 3D area where the fMRI scans would be done (i.e., whole brain) and adjusted the bounding box size and orientation of slice acquisition to each person’s AC-PC axis. A brief additional scan was also conducted to facilitate z-shim compensation^18^.

**Status–color association training.** As mentioned in the main text, participants completed a training task in the scanner before completing the impression formation task. In detail, the association training component involved three simple association training blocks^1^. In the first block, participants responded to 10 trials showing a silhouette superimposed on a colored background (viz., orange or blue: five per status level). Participants responded by tapping their right and left pointer fingers on the button-boxes. Each button-box was strapped to a glove that was worn on each hand. Each pointer finger represented one status level (high or low), and on each trial participants had to accurately identify what status level a given color represented. Each trial contributed to a cumulative accuracy score (*M* = 95.57% for all trials, *M* = 96.86% for the last five trials). Participants saw the cumulative accuracy score presented as feedback on the screen after each trial.

In the next block, the training flipped; participants responded to 10 trials (five per status level) asking them to accurately select the color that represents low or high status. The response key labels for low and high status were counterbalanced across participants. Each trial contributed to a cumulative accuracy score (*M* = 90.14% for all trials, *M* = 92% for the last five trials). Again, the cumulative accuracy score was presented as feedback on the screen. Upon the conclusion of this training and prior to any scanning, the experimenter verbally confirmed with participants that they could provide the correct color association for each level of status.

**Functional imaging experiments.** After the initial reference scans, participants completed two runs for an unrelated fMRI experiment on how people perceive video-recorded interactions between the police and civilians. This work is currently being prepared for publication, but a full description of this task is available upon request. Next, scanning paused while participants completed the status–color association training for the first time (details above and in main text). After training, scanning resumed for two consecutive runs of the impression formation task.

**Structural and resting-state scans.** After all functional images were acquired (described in main text), thin-slice resting-state scans were acquired using the following parameters: TR = 2,000 ms, TE = 29.5 ms, FOV = 240 × 138 × 240 mm, slice thickness = 2.6 mm with a 1.4-mm gap, an in-plane resolution of 3.75 mm^2^, and a flip angle of 77°. High-resolution structural images were subsequently acquired in the sagittal plane using a T1-weighted 3D Turbo Field Echo (TFE/MP-RAGE) anatomical scan (TR = 8.0 ms, TE = 3.5 ms, FOV = 228 × 240 × 181 mm, slice thickness = 1.0 mm without gaps, an in-plane resolution of 1 mm^2^, and a flip angle of 8°).

## Post-Scan Procedure

After scanning, participants completed several measures in the following order: the cop–civilian video rating survey, police contact questionnaire, evaluative priming task, likeability ratings, status recall, and the trust game (see Supplemental Text S1 for details). Finally, participants completed a block of questionnaires in the following order: state and trait anxiety, the PANAS, the single-item stress/anxiety scales, current and childhood contact questionnaires assessing exposure to men and women who vary in SES, modern sexism, ambivalent sexism, and feminist identification (see Supplemental Text S1 for details). After this block of surveys, participants were asked what they thought the studies were about, debriefed, and paid.

# S3: Supplemental Stimulus and Task Design Parameters

## Stimuli

Fourteen female and 14 male faces were selected from a pool of 75 female and male faces originally from the Kennedy Face Database^19^. Original photographs were from the shoulders up. Prior to pre-testing, the following inclusion criteria were used: (1) direct eye gaze, (2) upright head position, and (3) no glasses. All images were equated on contrast and luminance using the SHINE toolbox^20^, and backgrounds were changed to light gray. The resulting gray-scale pictures were cropped such that each contained only a face presented centrally on a 467 × 700 pixel frame. Finally, faces were superimposed on colored borders that served as learned cues of social status level (within each gender: seven orange and seven blue), resulting in a final picture size of 500 × 750 pixels.

To ensure that the resulting four groups were as similar as possible, faces were equated using ratings provided by the Kennedy Face Database and independent ratings collected on Amazon Mechanical Turk (MTurk). From the Kennedy Face Database, we pulled the actual age of all faces at the time they were photographed and the perceived age group in years for each face, which was rated by an independent sample (*n* = 108) on an eight-point-scale in the following increments: 1 = 18–24, 2 = 25–30, 3 = 31–40, 4 = 41–49, 5 = 50–59, 6 = 60–69, 7 = 70–79, and 8 = 80 years and older^19^. Separate groups of MTurk participants rated the pool of 75 faces (actual age 18- to 40-years-old) for gender (categorical and confidence judgments: *n* = 22), expression (emotion and intensity judgments: *n* = 22), attractiveness (*n* = 22), likeability (*n* = 24), and gender typicality (*n* = 22). For attractiveness, likeability, and gender typicality, ratings were provided on a seven-point scale (e.g., 1 = not trustworthy at all, 7 = very trustworthy). Twenty-two participants rated face gender using two measures for each face: (1) “What is the gender of the person pictured above?” (female or male), and (2) “How confident are you about the gender of the face?” Confidence was rated on a five-point scale from “not confident at all” to “very confident”. For participants judging emotional expression (*n* = 22), two ratings were given for each face: (1) “What is the facial expression of the person pictured above?” (Angry, Happy, Neutral, or Sad), and (2) “How intense is this facial expression?” Intensity was rated on a five-point scale from “not intense at all” to “very intense”. To be classified as a particular emotion, a face had to elicit at least 60% consensus among participants. Ambiguous expressions (e.g., happy-neutral and neutral-sad faces) were characterized by a total of at least 75% of classifications that were split between no more than two emotions (e.g., 35% neutral and 40% sad).

For each rating dimension, separate 2 (Target Gender: women, men) × 2 (Status Group: counterbalanced group 1, counterbalanced group 2) analyses of variance (ANOVA) ensured our stimuli sets were equated on most dimensions of interest. Each of the four stimulus groups was equated at approximately moderate attractiveness, *M* = 3.841, *F*(1,24) = 0.001, *p =* .978; and likeability, *M* = 4.083, *F*(1,24) = 0.048, *p =* .828; approximately 24 years old, *M* = 24.392, *F*(1,24) = 0.355, *p =* .557; and appearing to be approximately 25–30 years old on average, *M* = 2.320, *F*(1,24) = 0.874, *p =* .359. Each stimulus group was equated at low emotional intensity, *M* = 1.696, *F*(1,24) = 1.693, *p =* .205. The faces were categorized as having various emotional states by participants (happy, *n* = 8; neutral, *n* = 16; ambiguous, *n* = 4). We distributed these emotion categories evenly across the four stimulus sets: happy, *X^2^* (3, *n* = 8) = 0, *p =* .999; neutral, *X^2^* (3, *n* = 16) = 1, *p =* .801 and ambiguous, *X^2^* (3, *n* = 4) = 4, *p =* .261. Men’s faces were perceived as more gender typical than women’s faces, *F*(1,24) = 14.602, *p =* .001 (*M_Female_* = 4.41; *M_Male_* = 5.224), and people were more confident about the gender of men than the gender of women, *F*(1,24) = 24.845, *p <* .0001 (*M_Women_* = 3.942; *M_Men_* = 4.506). Nonetheless, all faces used as stimuli had 95% or greater consensus regarding target gender. Moreover, these effects of target face gender on perceived gender typicality and gender confidence were not reliably modulated by counterbalanced status group, *F*(1,24) < 0.030, *p >* .864, and *F*(1,24) < 0.921, *p >* .346, respectively.

## fMRI Trial Sequence Generation

Trial sequences for the impression-formation task were generated using optseq2^21^. The following parameters were entered: TR (repetition time) = 2, ntp (total time points) = 98, psdwin (post-stimulus delay window) = 0–20 seconds, four conditions (entered as events each with 14 occurrences of 2 seconds in duration), tnullmin (minimum null event duration) = 0 seconds, tnullmax (maximum null event duration) = 6 seconds. After 10,000 iterations, optseq returned several optimal trial sequences. The first two sequences not containing more than three consecutive repetitions of the same condition were selected for the two functional runs.

# **S4: Analyses of Exploratory Measures**

In this section, we report on results from (1) exploratory gender comparisons of subjective SES, objective SES, social dominance orientation, and impression formation response time; (2) exploratory analyses of pre- and post-scan measures assessing attitudes toward women and men that vary in SES, stimulus likeability, status recall, intergroup anxiety toward high- and low-SES men and women, intergroup contact with high- and low-SES men and women, modern sexism, and ambivalent sexism (i.e., hostile and benevolent sexism); and (3) exploratory ROI and whole-brain analyses that model the perceiver’s subjective status.

## Exploratory Gender Comparisons

To rule out some potential confounds of participant gender, we checked to see whether participants identifying as women and men differed on any potentially important variables related to social hierarchy and one’s position within the SES hierarchy.

**Subjective SES.** Two men in our sample failed to provide subjective status ratings and were subsequently removed from any analyses involving subjective status. (These two participants did report their gender and were therefore kept in the analyses described in the main text.) Overall, the subjective SES of our sample (n = 63) was above the MacArthur scale’s midpoint, *M* = 6.952, *SD* = 1.591, *Min* = 2, *Max* = 10. Although women trended lower in subjective status (*M* = 6.571, *SD* = 1.665) than men (*M* = 7.257, *SD* = 1.482), this difference was non-significant, *t_Welch_*(56.489) = -1.387, *p =* .171 (see Figure SF1). A chi-square test also indicated that the representation of women and men across the different levels of subjective status was not significantly different, *p* = .66. For the supplemental ROI analyses reported below, subjective status scores were standardized into *z* scores.


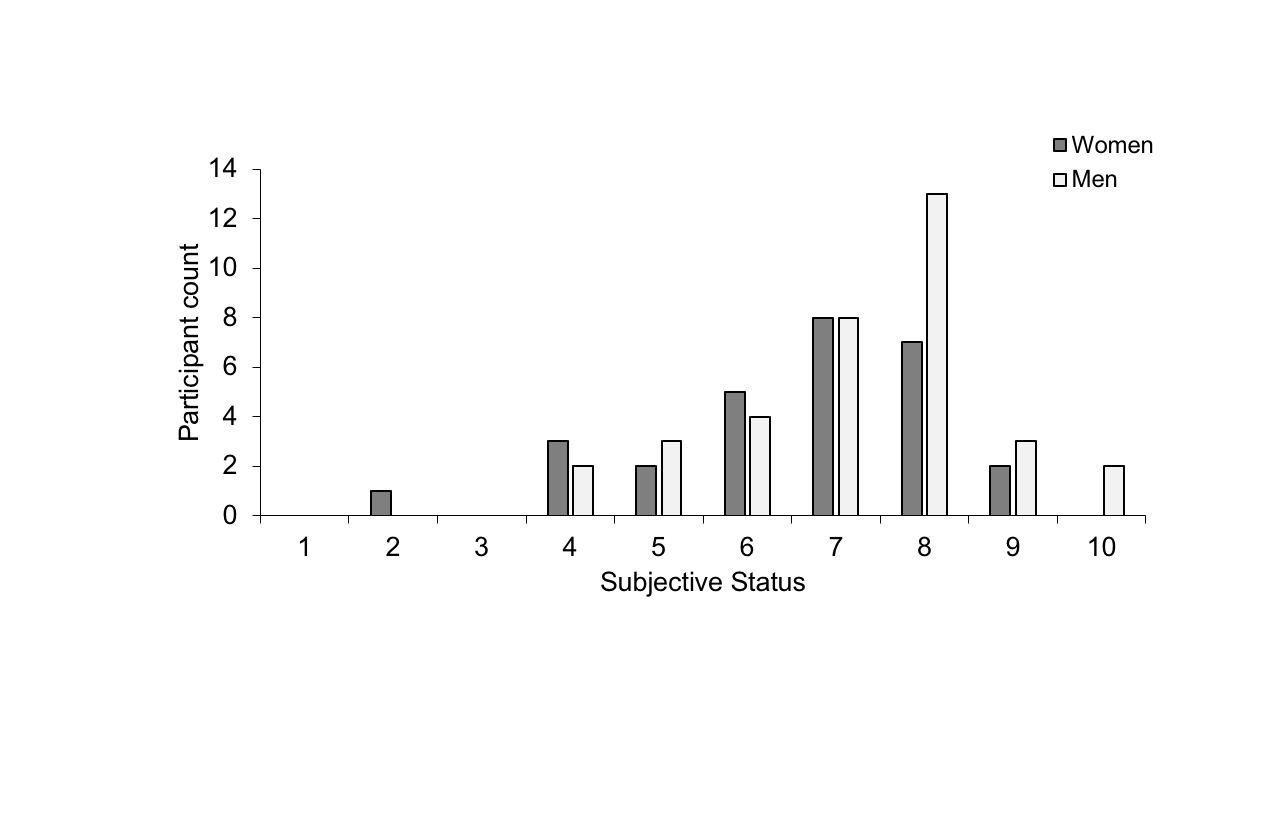


*Figure SF1.* Distribution of responses for subjective status plotted separately by participant gender. Subjective status was z-scored for supplemental ROI analyses but is plotted here as raw whole values for ease of interpretation.

**Objective SES.** Using self-report ratings of different measures of objective socioeconomic status, we explored potential perceiver gender differences that may contribute to the results from the main text. We found that in our sample, the representation of women and men was proportionally similar across levels of family income, *X^2^* (8, n = 56) = 10.263, *p* = .247; household savings, *X****^2^*** (8, n = 57) = 12.065, *p* = .148; household savings viability (i.e., how long it would take to go through one’s entire savings if unemployed), *X****^2^*** (4, n = 64) = 5.438, *p* = .245; net worth, *X****^2^*** (8, n = 57) = 5.600, p = .647; highest education, *X****^2^*** (6, n = 64) = 12.170, *p* = .058; parents’ family income, *X****^2^*** (9, n = 62) = 5.841, *p* = .756; and parents’ highest education, *X****^2^*** (3, n = 62) = 5.390, *p* = .250. Some participants did not respond to certain questions due to non-response or technical errors.

**Intergroup Anxiety.** We tested for differences as a function of perceiver gender in Intergroup Anxiety at the prospect of collaborating with high- and low-status men and women. A mixed linear model showed that a significant interaction between perceiver gender and the hypothetical collaborator’s gender predicted anticipated anxiety, *b* = -0.379, *SE* = 0.158, *CI_95%_* = [-0.688, -0.070], *t*(183.8) = -2.402, *p* = 0.017 (see Figure SF2). All other effects, including effects of hypothetical partner status, were non-significant, *p* > .22. Follow-up simple effects analyses on the significant two-way interaction indicated that women were more anxious than men about hypothetically working with men, *b* = -0.452, *SE* = 0.226, *CI_95%_* = [-0.895, -0.009], *t*(80.275) = -1.997, *p* = 0.049. All other simple effects were non-significant, *p* > .07.


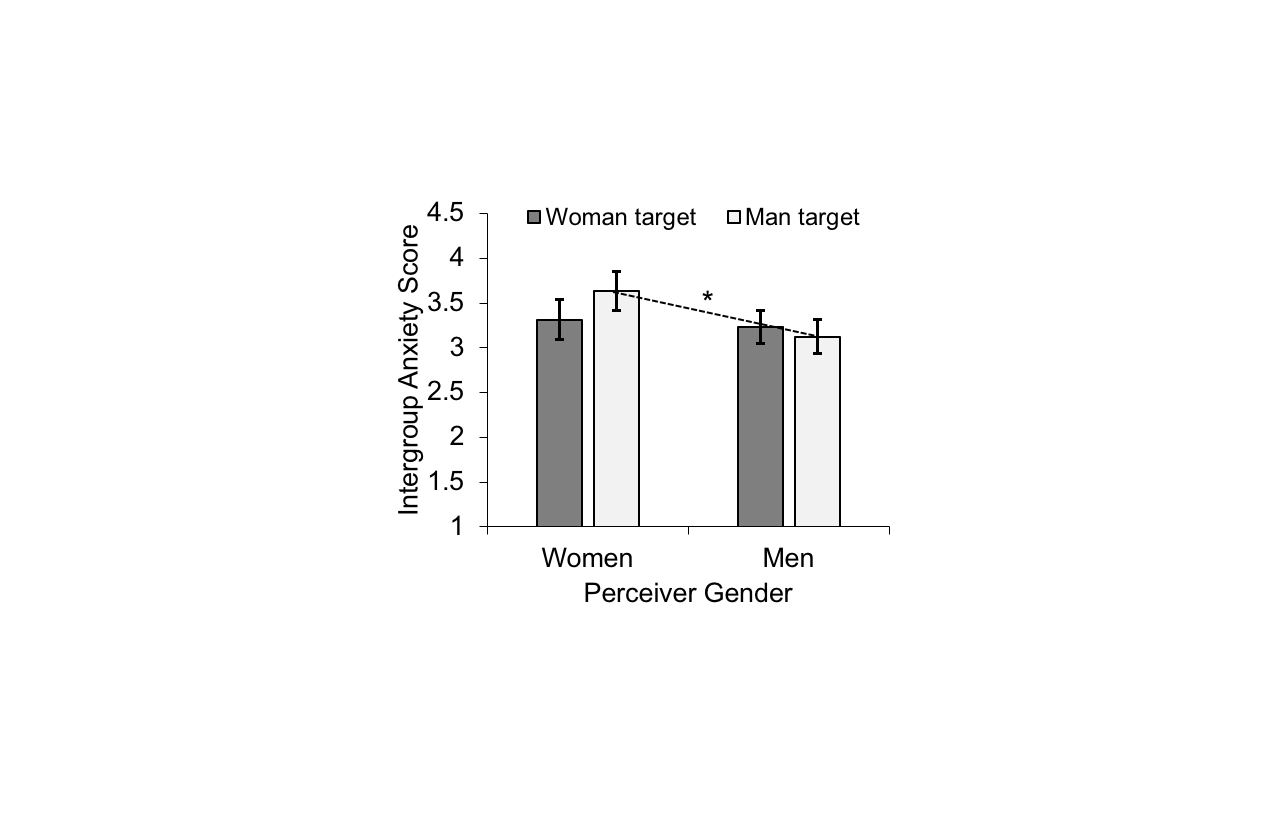


*Figure SF2.* Intergroup anxiety scores measuring anxiety at the prospect of collaborating with men and women (gray and white bars, respectively) for participants identifying as women (left) and men (right). Raw scores used for plotting whereas mean-centered scores were used for analysis. Error bars represent standard error. Significant simple effects are indicated, * = *p <* .05.

**Social dominance orientation.** We also tested for differences as a function of perceiver gender in social dominance orientation (SDO). Men (n = 34) and women (n = 27) did not differ in SDO, *t_welch_*(59) = -0.754, *p* = .454 (*M_Men_* = 2.075, *SD_Men_* = 0.955; *M_Women_* = 1.891, *SD_Women_* = 0.971).

**Feelings thermometers.** Using pre-scan feeling thermometer ratings of high- and low-status men and women in general, we tested for differences in attitude warmth as a function of the participant’s gender and four task conditions. This analysis included 62 participants (n_Women_ = 27, n_Men_ = 35) of the 65 participants from the main text analyses. Initially, six participants did not complete this section of the survey because of the addition of some survey measures after the first few subjects and because of catching an error in the presentation of the pre-test survey sections. Three subjects who experienced this error completed the unanswered sections after this error was fixed, whereas three subjects did not. We conducted a linear mixed-effects model predicting feeling thermometer ratings. The model included terms for a three-way interaction between target gender, target SES, and perceiver gender and all possible lower-order effects. We also modeled a random intercept by participant; no other random effects were modeled. Results revealed a main effect, such that women overall were rated as warmer than men, *b* = -13.273, *SE* = 2.196, *CI_95%_* = [-17.577, -8.969,], *t*(180) = -6,046, *p* < .001. This effect was moderated by perceiver gender in a two-way interaction, *b* = 9.861, *SE* = 4.391, *CI_95%_* = [1.255, 18.467], *t*(180) = 2.246, *p =* .026 (see Figure SF3). All other effects were non-significant, *p >* .24.

We followed up on this significant interaction with simple effects analyses between and within participant gender groups (see Table SF3). Men’s attitudes were warmer than women’s attitudes toward men in general as indicated by a significant main effect of perceiver gender when evaluating men in general, *b* = 0.425, *SE* = 0.205, *CI_95%_* = [0.0232, 0.827], *t*(109.16) = 2.076, *p* = .040. All other simple effects were non-significant, *p >* .09.


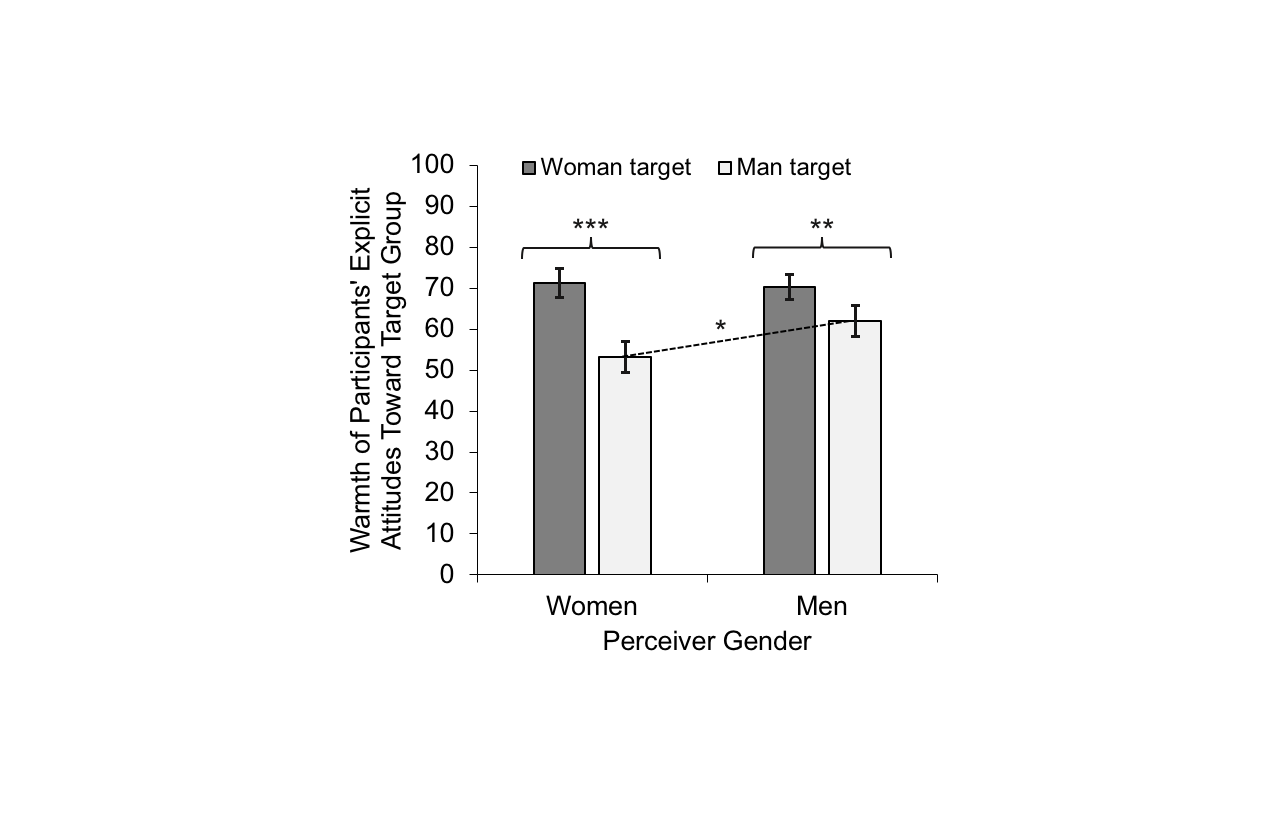


*Figure SF3.* Feelings thermometer scores measuring warmth as a function of target gender for participants identifying as women (left) and men (right). Raw scores used for plotting whereas mean-centered scores were used for analysis. Error bars represent standard error. Significant simple effects are indicated, * = *p <* .05, ** = *p <* .01, *** = *p <* .001.


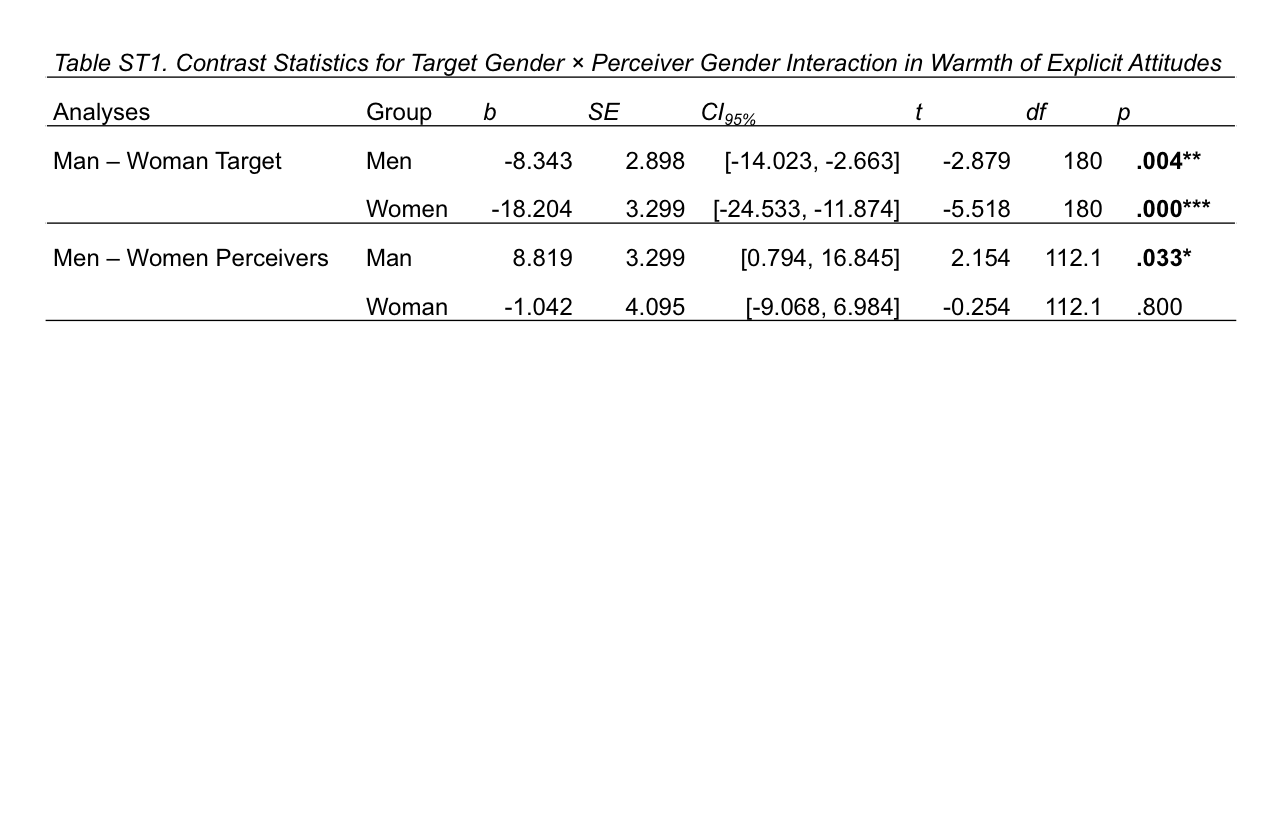


**Impression formation RTs.** Bearing in mind that the response time (RT) data from the impression formation task were truncated at 1,500 ms^[[1]](#footnote-1)^, we tested for gender differences in the time it took for participants to report that they formed their impressions. A mixed linear model indicated that the only effect that approached significance was a three-way interaction between perceiver gender, target SES, and target gender, *p* = .064. All other effects of perceiver gender, target SES, and target gender were non-significant, *p* > .19. Additional simple comparisons confirmed that women and men showed no significant differences in impression formation response times for any of the four face conditions, *p*> .34.

**Stimulus likeability.** Using post-scan likeability ratings of the face stimuli used in the impression formation fMRI task, we tested for any differences in likeability as a function of the participant’s gender and the four task conditions. Two participants did not complete this task due to technical difficulties, resulting in a sample size of 63 for this analysis. A linear model testing for a three-way interaction between target gender, target status, and perceiver gender (and all possible lower-order effects) on post-scan likeability ratings showed a significant main effect of gender, *b* = -0.314, *SE* = 0.071, *CI_95%_* = [0.175, 0.453], *t*(61.1) = -4.441, *p <* .001. Despite our efforts to equate the stimuli on likeability (see Supplemental Text S3), participants rated women targets as more likeable than men targets. All other effects were non-significant, *p >* .112. This finding is consistent with explicit gender-based stereotypes and attitudes that characterize women as warmer than men^22,23^.

**Status recall.** In the post-scan status recall task, participants saw the faces of men and women that were used as stimuli during the impression formation fMRI task, but this time without their colored backgrounds that indicated status levels. One participant did not complete this task due to technical difficulties, resulting in a sample size of 64. As an added check on whether participants were spontaneously attending to and learning the status information we presented together with each face, we conducted a logistic regression testing for a three-way interaction between target gender, target status, and perceiver gender (and all possible lower-order effects) on participants’ ability to correctly recall the status level for each face. Overall, participants correctly recalled the status of faces 65.5% of the time, which was significantly above chance, *b* = 0.721, *SE* = 0.102, *CI_95%_* = [0.521, 0.921], *z* = 7.037, *p* < .0001. All effects from the logistic regression were non-significant, *p >* .062. Taken together, results from the status recall task suggest that participants managed to learn the status of some faces presented during the impression formation task, even though they were not asked to memorize status information beyond the status-color associations introduced at the start of the task. This provides some indirect evidence that participants were attending to status when they formed their impressions in the scanner.

**Contact with low- and high-SES men and women.** In the final post-scan survey, participants completed a contact survey where they provided percentages of how many high, medium, and low-SES people they encountered in their childhood (age 0–18) and currently. They also provided percentages of how many of these individuals were women and men. To assess whether men and women in our sample had different levels of contact with high- and low-SES men and women during different life stages, we ran a mixed linear model to test the interaction between target gender, target SES, perceiver gender, and life stage on percentage of contact. Two two-way interactions (Life Stage × Target SES and Target Gender × Target SES) and main effects of life stage, target SES, and target gender were modeled as random effects. The results revealed a significant main effect of SES level, *b* = 0.570, *SE* = 0.147, *CI_95%_* = [0.282, 0.858], *t*(60) = 3.877, *p* < .001. Participants overall indicated greater contact with high-SES individuals than low-SES individuals. We also observed a significant main effect of target gender, *b* = 0.145, *SE* = 0.054, *CI_95%_* = [0.038, 0.252], *t*(61) = 2.668, *p* = .010. Participants indicated they had greater contact with men than women. We also observed a significant interaction between target SES and life stage, *b* = 0.428, *SE* = 0.168, *CI_95%_* = [0.098, 0.758], *t*(60) = 2.544, *p* = .014 (see Figure SF4). All other effects were non-significant, *p* > .12.

Following up on the significant interaction between target SES and life stage, we found a significant simple effect of SES in childhood, *b* = 0.356, *SE* = 0.158, *CI_95%_* = [0.047, 0.665], *t*(61) = 2.26, *p* = .027; a significant simple effect of SES in current life stage, *b* = 0.784, *SE* = 0.180, *CI_95%_* = [0.430, 1.138], *t*(60) = 4.346, *p* < .001; and a significant simple effect of life stage for high-SES individuals, *b* = 0.243, *SE* = 0.118, *CI_95%_* = [0.011, 0.475], *t*(61) = 2.057, *p* = .044. All other effects were non-significant, *p* > .137. These results suggest that participants overall reported greater contact with high-SES individuals relative to low-SES individuals in childhood and currently; and furthermore, reported significantly more contact with high-SES individuals currently than in childhood. Although these results show promise in understanding the relationship between life experience and status-based perception, the measure they are derived from is exploratory. Further testing is needed to explore its validity.

**
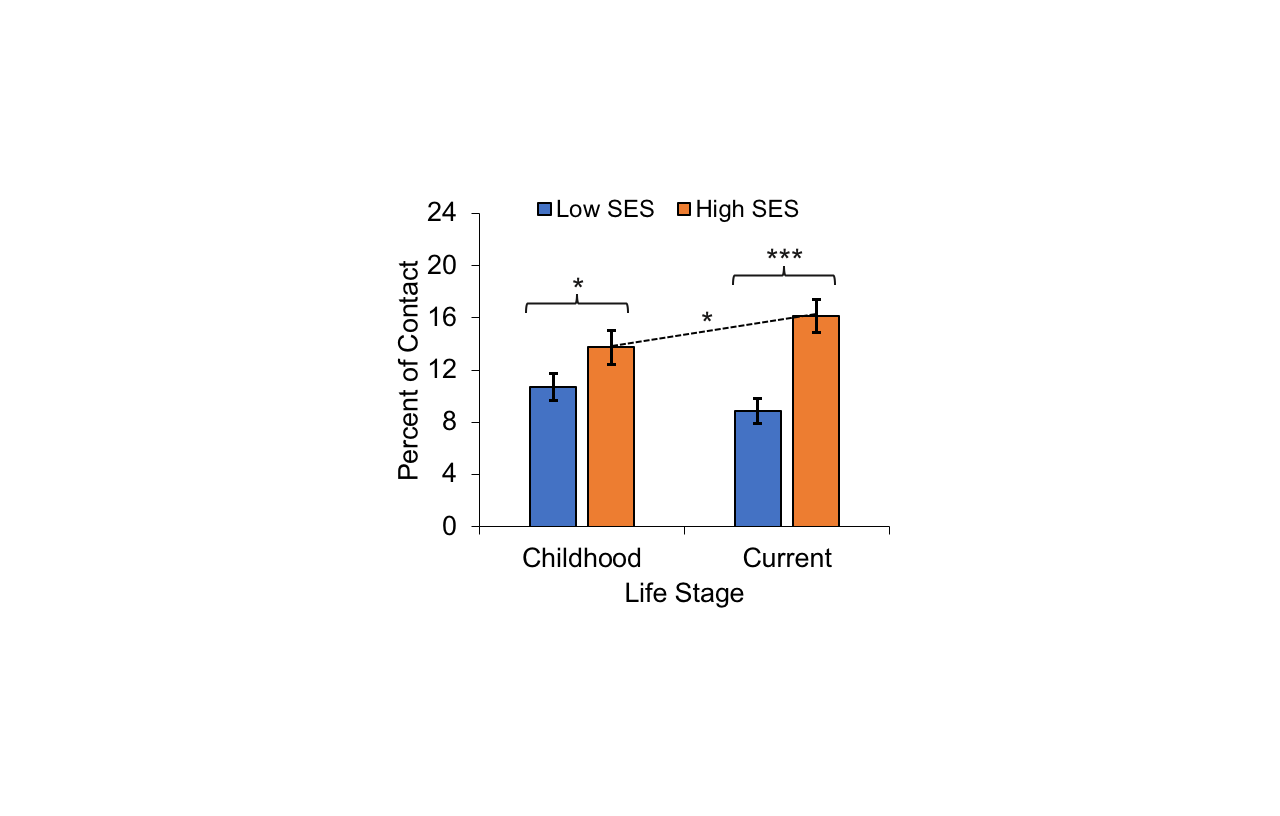
**

*Figure SF4.* Scores representing the percent of contact with high- and low-SES individuals in childhood and adulthood/currently. Raw percentage scores here are used for plotting whereas mean-centered z-scores were used for analysis. Error bars represent standard error. Significant simple effects are indicated, * = *p* < .05, *** = *p <* .001.

**Modern and Ambivalent sexism.** Men and women did not significantly differ on explicit ratings of modern sexism (*p* > .45); hostile sexism *(p* > .54); or benevolent sexism (*p* > .93). Our participants were on average slightly below the scale midpoint for modern sexism (*M* = 2.228, *SD* = 0.573); hostile sexism (*M* = 2.351, *SD* = 0.967); and benevolent sexism (*M* = 2.808, *SD* = 0.846).

## Supplemental ROI Analyses Including Subjective Status

Our primary analyses (see main text) focused on the relationship between perceiver gender and task-related activity in key regions of interest (ROI). For the sake of comprehensiveness, we also report here supplemental ROI analyses that test the robustness of effects reported in the main text when effects of the perceiver’s subjective status are also included in the model. All ROI analyses were completed using lme4 package for linear mixed-effects models^24^ in R^25^. Degrees of freedom were estimated using Satterthwaite’s approximation, provided by the package lmerTest, version 2.0-36^26^. Separate ROI analyses were conducted for the right NAcc, left NAcc, VMPFC, right amygdala, and left amygdala (see main text for details on ROI selection, volume, and coordinates). Therefore, we used Bonferroni correction to adjust the alpha level to .01 for each omnibus model. For each ROI, linear mixed-effects models predicted average BOLD signal as a function of the perceiver’s subjective SES and gender and the target’s SES and gender. A random intercept for participant-level variation was also included.

**NAcc.** In the right NAcc, we observed a significant Perceiver Gender × Target SES interaction, *b* = 0.392, *SE* = 0.120, *CI_95%_* = [0.156, 0.630], *t*(177) = 3.259, *p =* .001, as in the main text (Figure 2B). We also observed a Target Gender × Subjective SES interaction, *b* = 0.127, *SE* = 0.058, *CI_95%_* = [0.013, 0.241], *t*(177) = 2.176, *p =* .031, but this did not survive Bonferroni correction. All other effects in the right NAcc and left NAcc were non-significant (*p* > .125 and *p* > .081, respectively).

***Target SES × Perceiver Gender interaction in the right NAcc.*** A series of follow-up models tested for: (1) the simple effects of target SES for men (men = 0, women = 1) and women (men = 1, women = 0), and (2) the simple slopes of perceiver gender for high (high = 0, low = 1) and low SES (low = 0, high = 1). Collapsing across target gender (by using standard contrast coding: +/- 0.5) and subjective SES (by using mean-centered z-scores), these models predicted right NAcc response as a function of perceiver gender and target SES. The right NAcc parameter estimates resulting from these follow-up models are plotted in Figure SF5 and the simple effects statistics are in Table ST2. The simple slopes for low and high target SES were non-significant, *p >* .080. Consistent with the main text (see Table 1), men showed more activity in the right NAcc in response to high- than low-SES targets, *p =* .014. This simple effect was also significant in the opposite direction for women, *p =* .033.

*
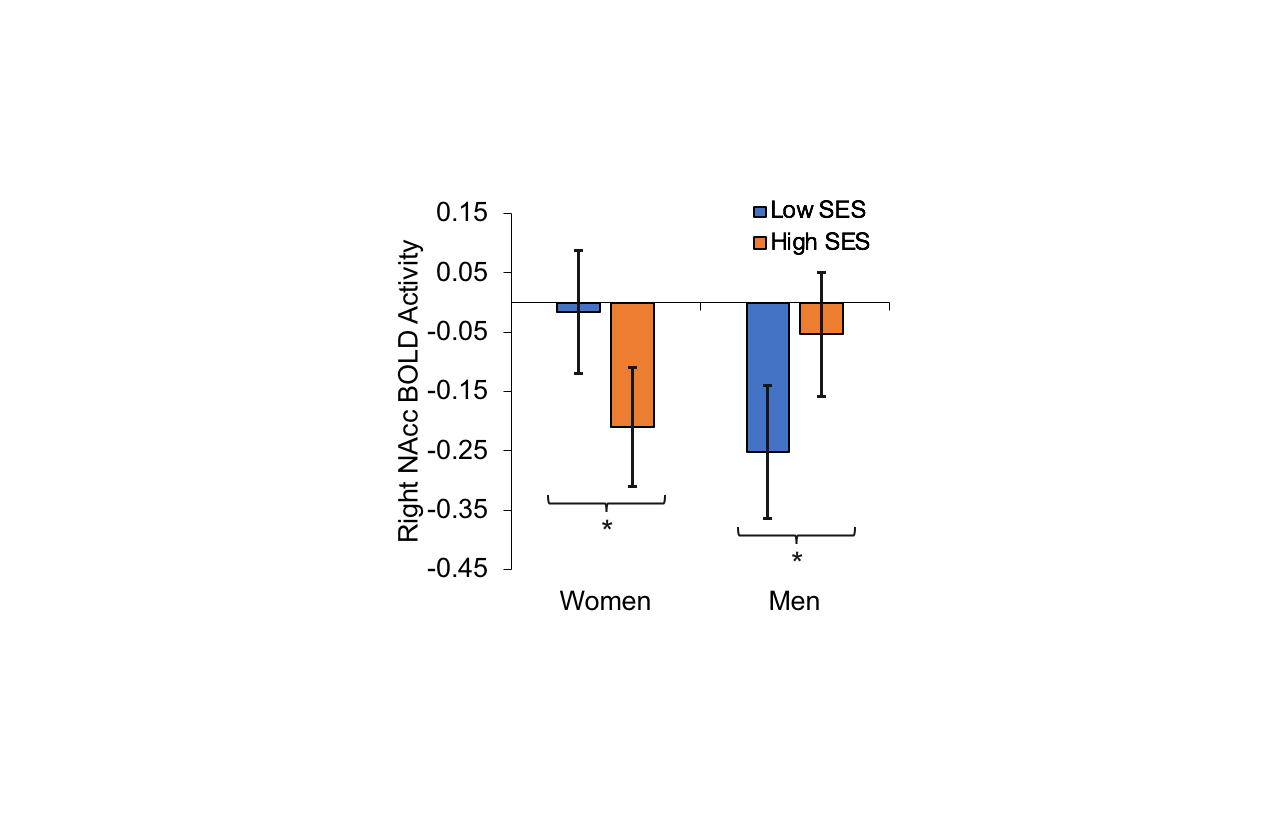
*

*Figure SF5.* Right NAcc response as a function of target SES for participants identifying as women (left) and men (right). Significant simple effects (*) are indicated, p < .05.

***
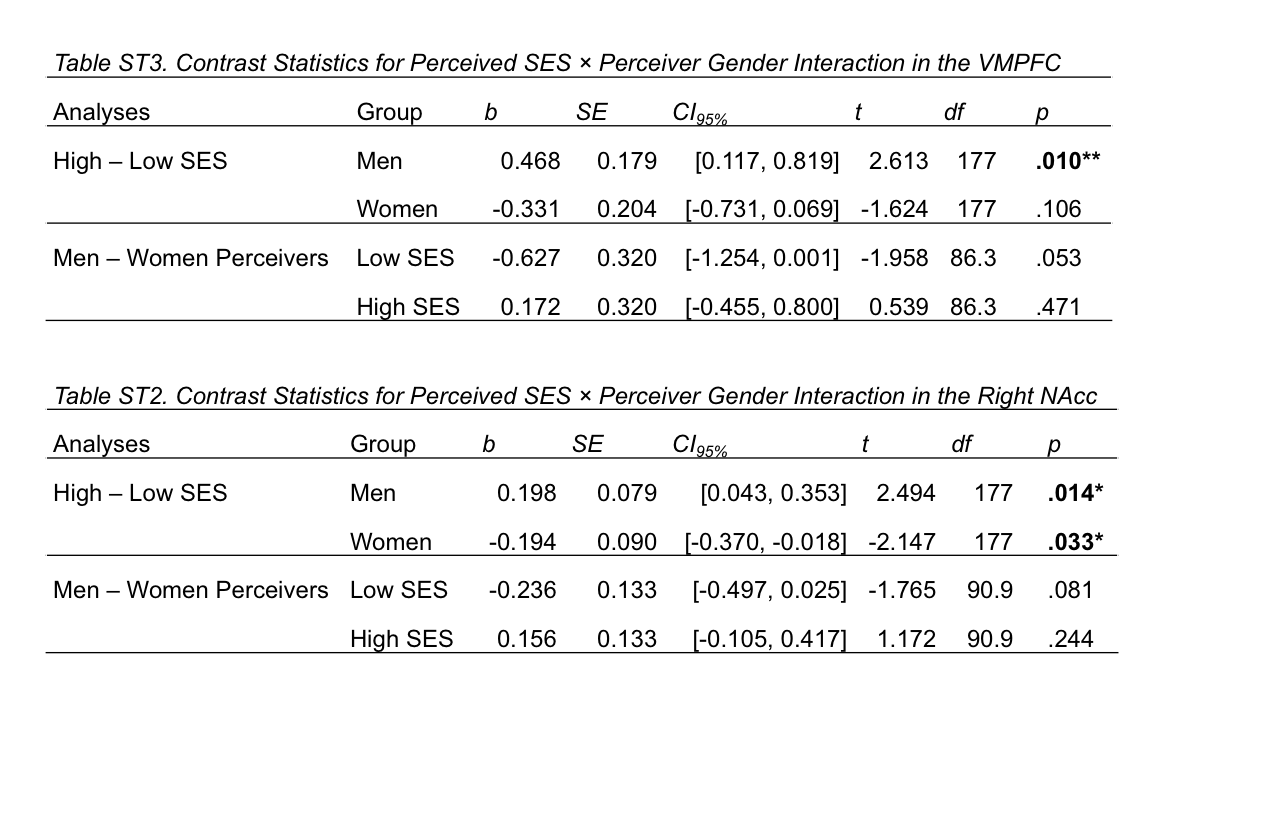
***

**VMPFC.** In the VMPFC, we observed a significant Target SES × Perceiver Gender interaction consistent with our finding in the main text (Figure 2A), *b* = 0.799, SE = 0.272, *CI_95%_* = [0.267, 1.331], *t*(180) = 2.690, *p =* .008. All other analyses in this region failed to yield any additional significant effects (all *p* > .244).

***Target SES* × *Perceiver Gender interaction in the VMPFC.*** A series of follow-up models tested for: (1) the simple effects of target SES for male (male = 0, female = 1) and female perceivers (male = 1, female = 0), and (2) the simple slopes of perceiver gender for high (high = 0, low = 1) and low SES (low = 0, high = 1). Collapsing across target gender (by using standard contrast coding: +/- 0.5) and subjective SES (by using mean-centered z-scores), these models predicted VMPFC response as a function of perceiver gender and target SES. The VMPFC parameter estimates resulting from these follow-up models are plotted in Figure SF6 and the simple effects statistics are in Table ST3. The simple slopes of target SES were both non-significant, *p >* .052. Men showed significantly more VMPFC activity when forming impressions of high-SES (vs. low-SES) targets, *p =* .010. Women did not show a significant effect of target SES, *p =* .106.


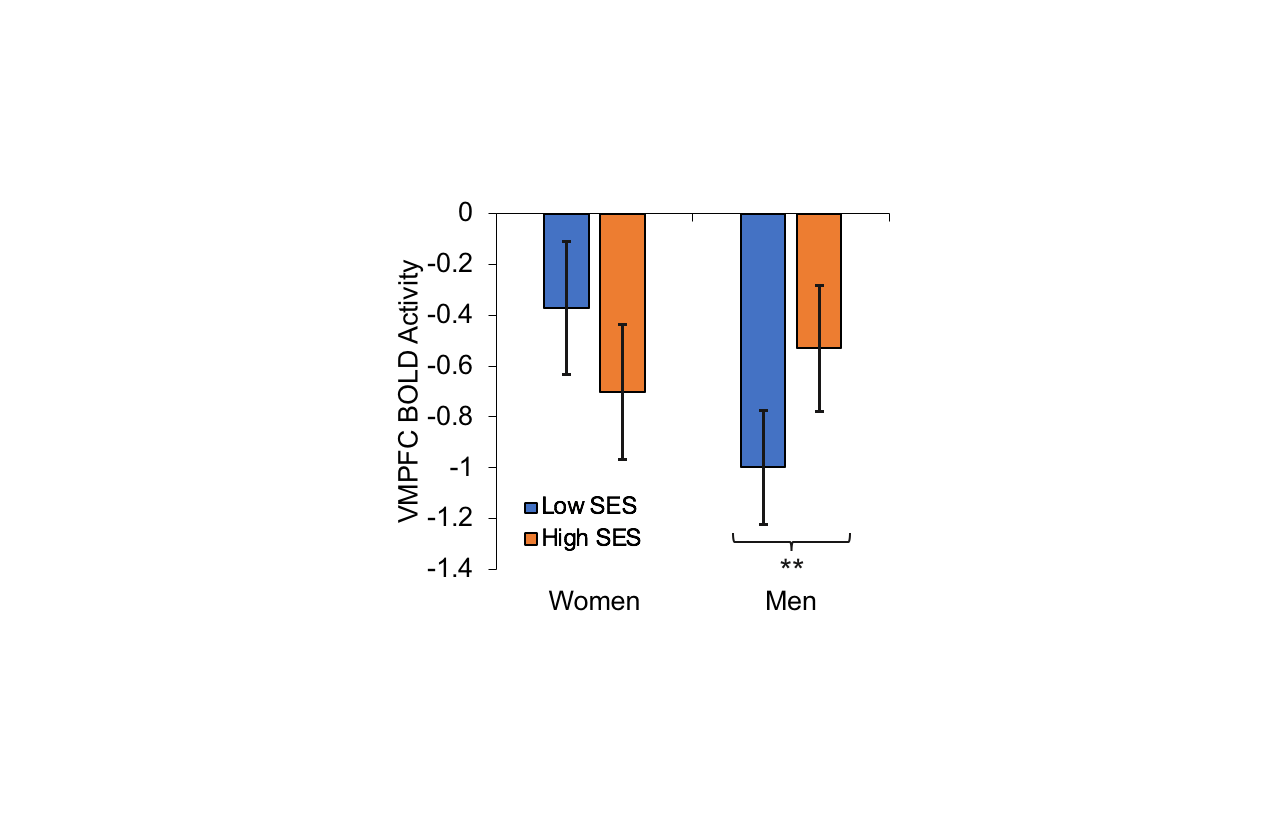


*Figure SF6.* VMPFC response as a function of target SES for women (left) and men (right). Significant simple effects (**) are indicated, p < .01.

***
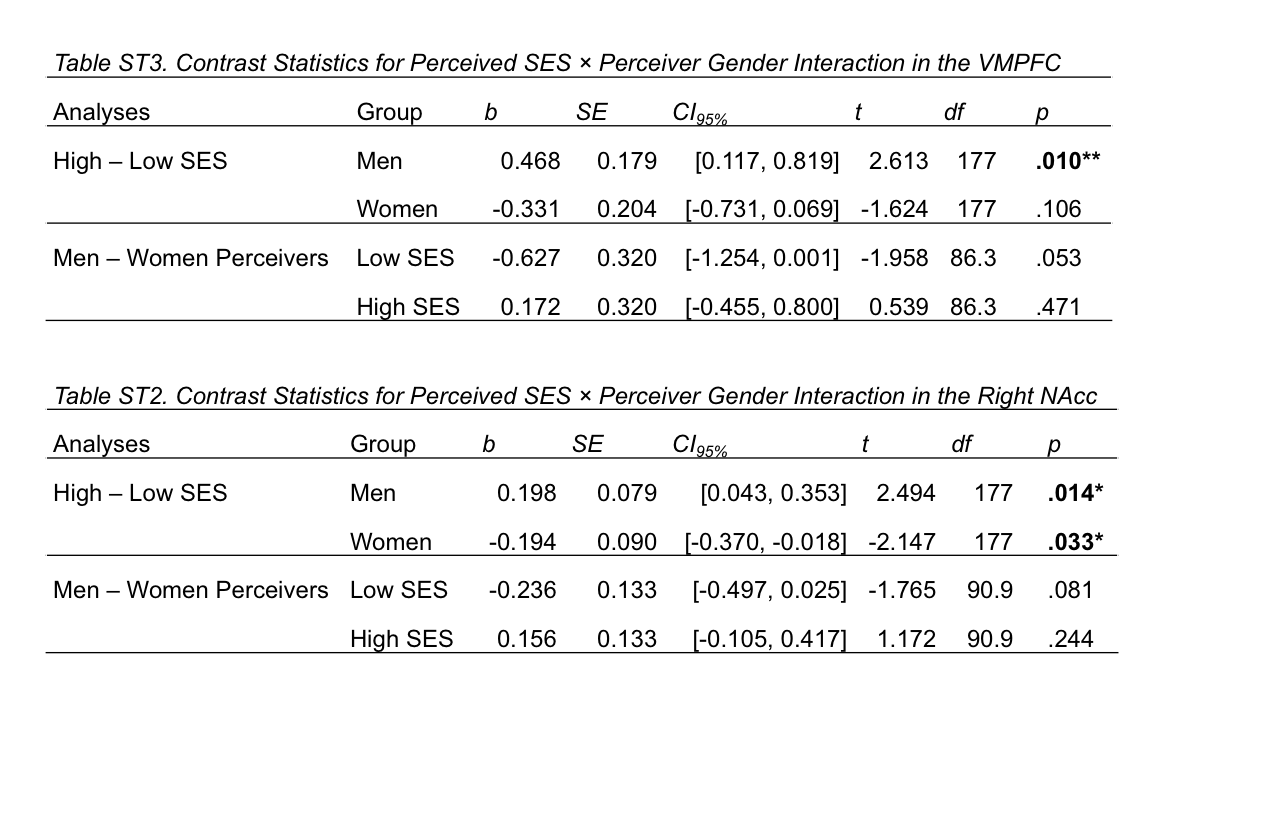
***

**Amygdala.** The left amygdala showed a Target Gender × Perceiver Gender interaction, *b* = 0.272, SE = 0.129, *CI_95%_* = [-0.347, -0.030], *t*(177) = 2.109, *p =* .036, however this did not survive Bonferroni correction. The right amygdala showed a main effect of target gender, *b* = -0.188, *SE* = 0.081, *CI_95%_* = [0.267, 1.331], *t*(177) = -2.328, *p =* .021; that similarly did not survive Bonferroni correction. All other analyses in the left (*p* > .203) and right amygdala failed to yield any additional significant effects (*p* > .072).

**Summary of supplemental ROI analyses*.*** When including subjective SES in the models reported in the main text, the preferential NAcc and VMPFC activity that men showed for high (vs. low) SES targets remained robust when all possible effects of perceiver subjective SES were included in the model.

## Supplemental Seed PLS Analyses

To supplement the seed PLS analysis in the main text, we conducted separate seed PLS analyses for men (Figure SF7) and women perceivers (Figure SF8). Intriguingly, the results from the seed PLS analysis in the main text were similar for both gender subsamples (see Tables ST4 and ST5). Both analyses revealed significant LVs (*p* < .001) illustrating significant relationships between the average VMPFC response to high (vs. low) SES (i.e., the seed) and co-activation of an extended network in level-1 high SES > low SES univariate contrast images. This suggests that women and men recruit similar processes when forming impressions of people varying in SES, but men in the context of our task tended to recruit those neural pathways more by virtue of their overall higher responses to high (vs. low) SES in the VMPFC (see ROI analyses reported in the main text).


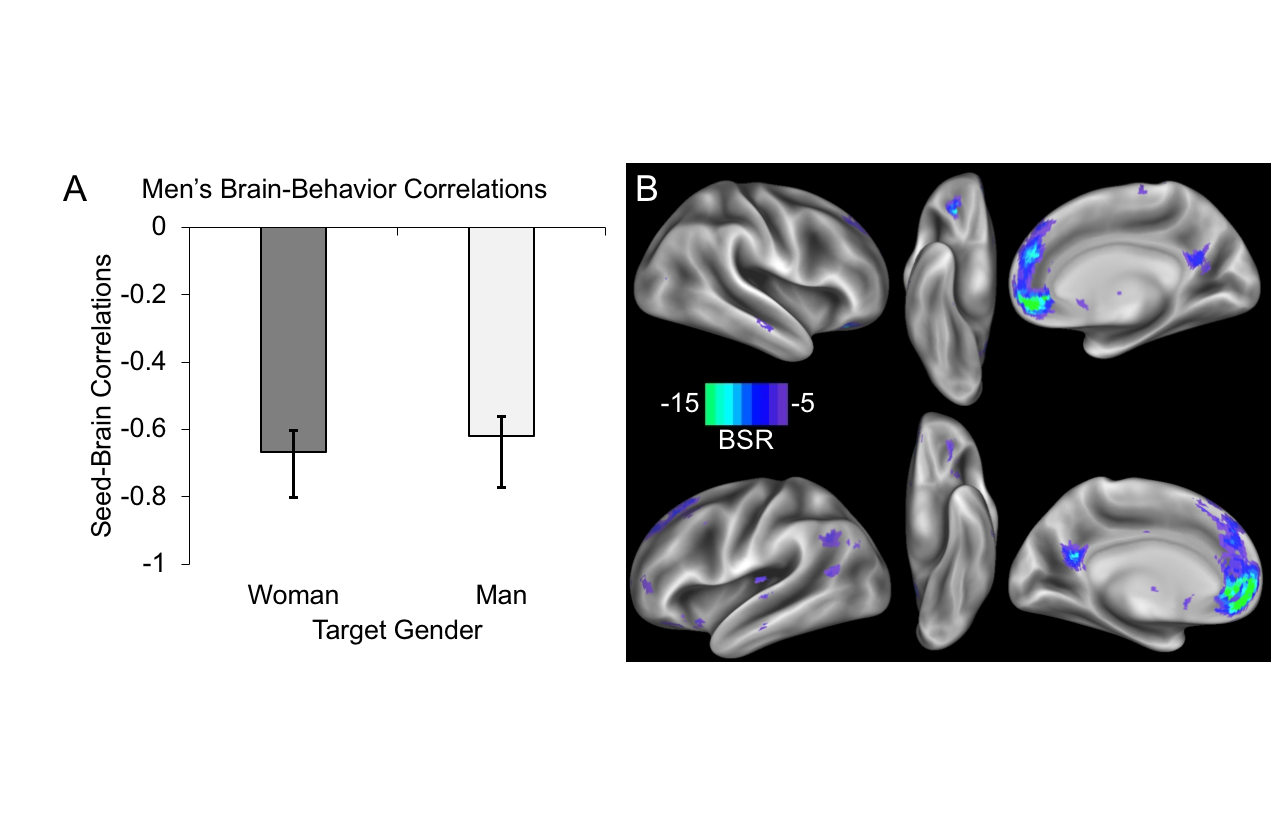


*Figure SF7.* (A) Across both target genders, a significant latent variable emerged for men that captured a relationship between average responses to high (vs. low) SES in the VMPFC ROI seed and voxel co-activation in level-1 contrast images reflecting high SES > low SES. Seed-brain correlations plotted on the y-axis represent the strength of the relationship captured by the latent variable separately for faces depicting women and men. The error bars represent confidence intervals computed through a 2000-sample bootstrapping procedure. (B) Patterns of whole-brain co-activation that most strongly contribute to the latent variable. Lateral, ventral, and medial views are displayed separately for the right hemisphere (top three images) and left hemisphere (bottom three images). Voxels were thresholded at BSR ≤ -5. Note that the directionality of brain co-activation (B) needs to be interpreted in conjunction with the bar graph (A); lighter colored voxels indicate greater co-activation as a function of increasingly preferential VMPFC responses to high versus low SES.


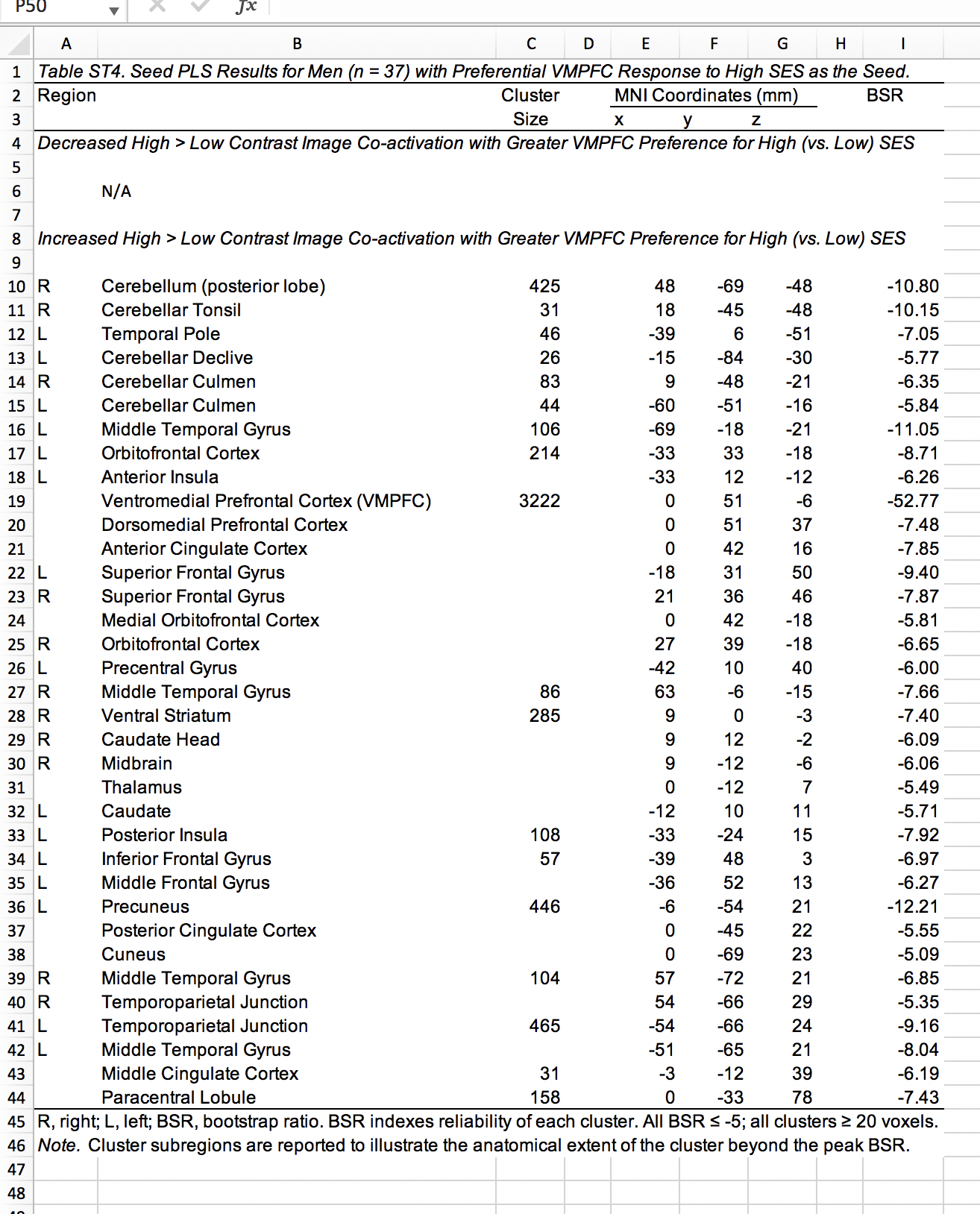


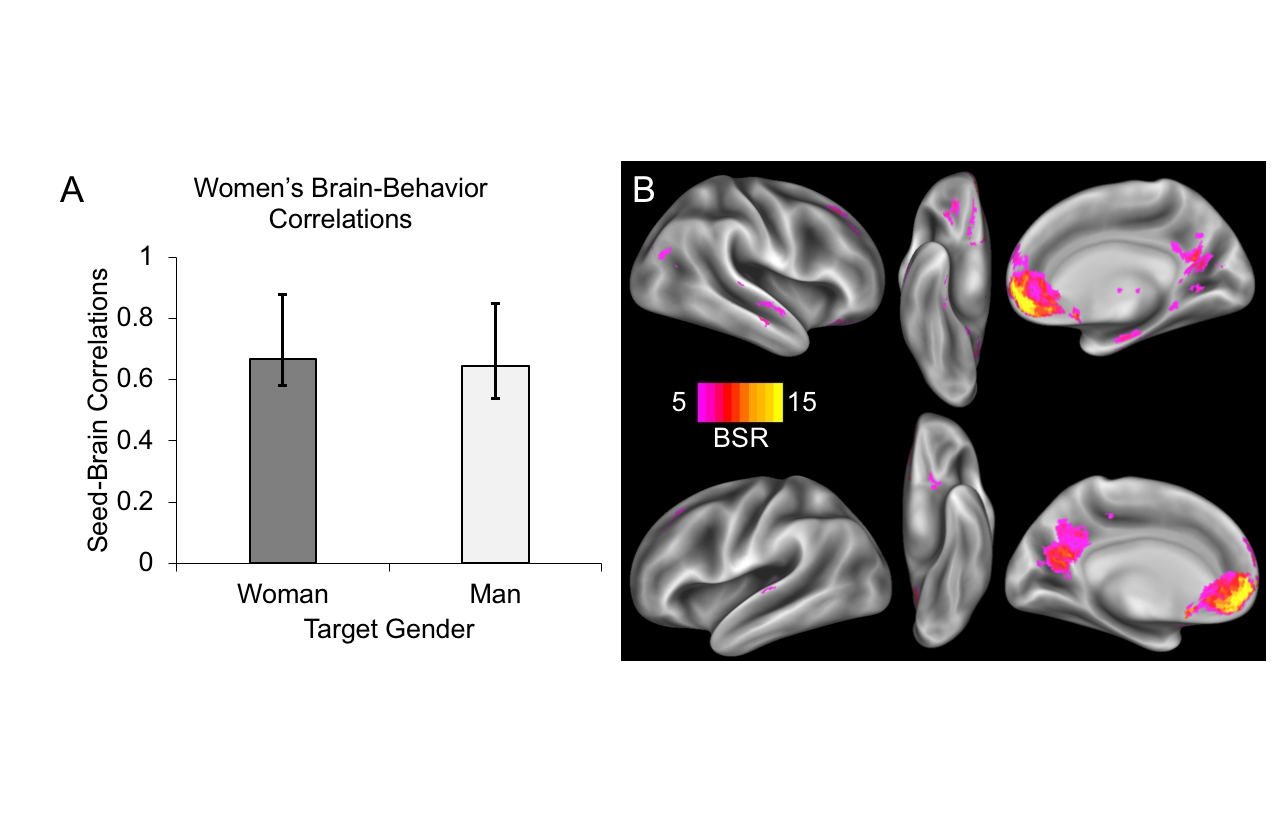


*Figure SF8.* (A) Across both target genders, a significant latent variable emerged for women that captured a relationship between average responses to high (vs. low) SES in the VMPFC ROI seed and voxel co-activation in level-1 contrast images reflecting high SES > low SES. Seed-brain correlations plotted on the y-axis represent the strength of the relationship captured by the latent variable separately for faces depicting women and men. The error bars represent confidence intervals computed through a 2000-sample bootstrapping procedure. (B) Patterns of whole-brain co-activation that most strongly contribute to the latent variable. Lateral, ventral, and medial views are displayed separately for the right hemisphere (top three images) and left hemisphere (bottom three images). Voxels were thresholded at BSR ≥ 5. Note that the directionality of brain co-activation (B) needs to be interpreted in conjunction with the bar graph (A); lighter colored voxels indicate greater co-activation as a function of increasingly preferential VMPFC responses to high versus low SES.


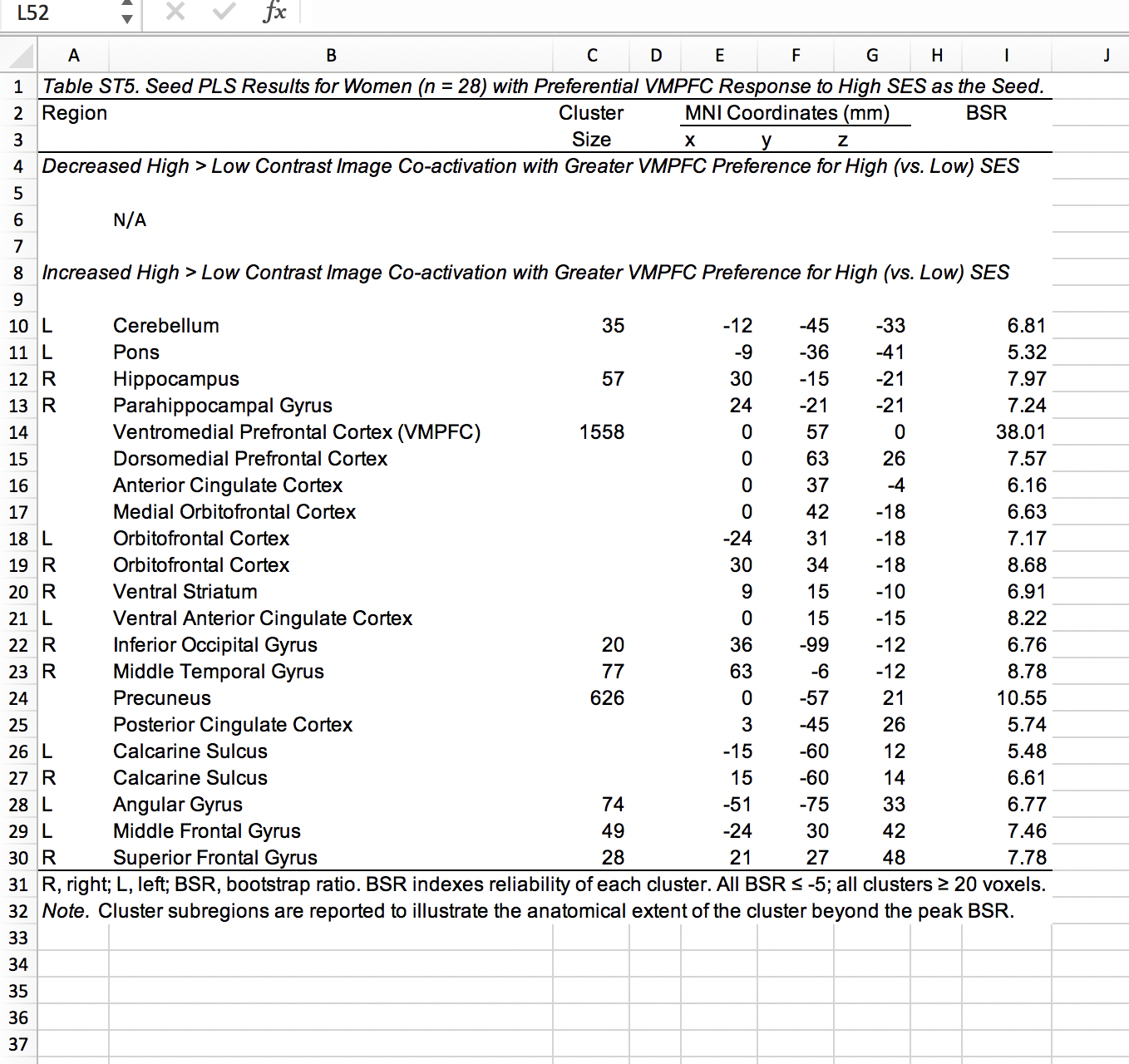


# References

1. Mattan, B. D., Kubota, J. T., Dang, T. P. & Cloutier, J. External motivation to avoid prejudice alters neural responses to targets varying in race and status. *Soc. Cogn. Affect. Neurosci.* **13**, 22–31 (2018).

2. Oakes, J. M. & Rossi, P. H. The measurement of SES in health research: Current practice and steps toward a new approach. *Soc. Sci. Med.* **56**, 769–784 (2003).

3. Adler, N. E., Epel, E. S., Castellazzo, G. & Ickovics, J. R. Relationship of subjective and objective social status with psychological and physiological functioning: Preliminary data in healthy white women. *Heal. Psychol.* **19**, 586–592 (2000).

4. Pratto, F., Sidanius, J., Stallworth, L. M. & Malle, B. F. Social dominance orientation: A personality variable predicting social and political attitudes. *J. Pers. Soc. Psychol.* **67**, 741–763 (1994).

5. Greenland, K., Xenias, D. & Maio, G. Intergroup anxiety from the self and other: Evidence from self-report, physiological effects, and real interactions. *Eur. J. Soc. Psychol.* **42**, 150–163 (2012).

6. Spielberger, C. D., Gorsuch, R. L., Lushene, R., Vagg, P. R. & Jacobs, G. A. *Manual for the State-Trait Anxiety Inventory*. (Consulting Psychologists Press, 1983).

7. Watson, D., Clark, L. A. & Tellegen, A. Development and validation of brief measures of positive and negative affect: The PANAS scales. **54**, 1063–1070 (1988).

8. Skogan, W. G. Asymmetry in the Impact of Encounters with Police. *Polic. Soc.* **16**, 99–126 (2006).

9. Mattan, B. D., Kubota, J. T., Li, T., Venezia, S. A. & Cloutier, J. Implicit evaluative biases toward targets varying in race and socioeconomic status. *Personal. Soc. Psychol. Bull.* **45**, 1512–1527 (2019).

10. FeldmanHall, O., Raio, C. M., Kubota, J. T., Seiler, M. G. & Phelps, E. A. The effects of social context and acute stress on decision-making under uncertainty. *Psychol. Sci.* **26**, 1918–1926 (2015).

11. Cloutier, J., Li, T. & Correll, J. The impact of childhood experience on amygdala response to perceptually familiar Black and White faces. *J. Cogn. Neurosci.* **26**, 1992–2004 (2014).

12. Pettigrew, T. F. Generalized intergroup contact effects on prejudice. *Personal. Soc. Psychol. Bull.* **23**, 173–185 (1997).

13. Swim, J. K., Aikin, K. J., Hall, W. S. & Hunter, B. A. Sexism and racism: Old-fashioned and modern prejudices. *J. Pers. Soc. Psychol.* **68**, 199–214 (1995).

14. Glick, P. & Fiske, S. T. The ambivalent sexism inventory: Differentiating hostile and benevolent sexism. **70**, 491–512 (1996).

15. Psychology Software Tools Inc. E-Prime 3.0. (2017).

16. Millisecond Software. Inquisit 5. (2016).

17. Qualtrics. Qualtrics: The World’s Leading Research & Insights Platform. *Qualtrics* (2016) doi:10.3109/03639045.2013.790903.

18. Du, Y. P., Dalwani, M., Wiley, K., Claus, E. & Tregellas, J. R. Reducing susceptibility artifacts in fMRI using volume-selective z-shim compensation. *Magn. Reson. Med.* **57**, 396–404 (2007).

19. Kennedy, K. M., Hope, K. & Raz, N. Life span adult faces: Norms for age, familiarity, memorability, mood, and picture quality. *Exp. Aging Res.* **35**, 268–275 (2009).

20. Willenbockel, V. *et al.* Controlling low-level image properties: The SHINE toolbox. *Behav. Res. Methods* **42**, 671–684 (2010).

21. Greve, D. N. Optseq2. (2002).

22. Fiske, S. T., Cuddy, A. J. C., Glick, P. & Xu, J. A model of (often mixed) stereotype content: Competence and warmth respectively follow from perceived status and competition. **82**, 878–902 (2002).

23. Eagly, A. H. & Mladanic, A. Gender stereotypes and attitudes toward women and men. *Personal. Soc. Psychol. Bull.* **15**, 543–558 (1989).

24. Bates, D., Maechler, B., Bolker, B. & Walker, S. Fitting linear mixed-effects models using lme4. *J. Stat. Softw.* **67**, 1–48 (2015).

25. R Core Team. R: A language and environment for statistical computing. (2017).

26. Kuznetsova, A., Brockhoff, P. B. & Christensen, R. H. B. lmerTest Package: Tests in Linear Mixed Effects Models. *J. Stat. Softw.* **82**, (2017).

1. The impression formation task was programmed to record RTs only for responses made within 1500 ms, which is when the face stimulus disappeared. Thus, non-responses or responses after 1500 ms were both recorded as 0. Given the above limitations, we looked at participants’ button press patterns to help identify excessive careless responses. Button press responses were recorded between 9%–100% of the time for all participants (*M* = 93.75%, *SD* = 16.89%). Participants on average responded to experimental trials at 860 ms (*SD* = 182 ms). Based on these group averages, we removed one participant outlier who had an unusually low response rate (9%) which is described in the Method section of the main text. This participant’s completed responses were also rather fast for an impression formation task: *M_RT_* = 381.40 ms and *SD_RT_* = 407.13 ms. Data from all other participants were retained for analysis on the presumption that many unrecorded responses were more likely due to response latencies exceeding 1500 ms than to failures to respond at all. For example, the participant with the lowest response rate after excluding the outlier had a 64% response rate. Closer inspection of the data revealed that their response times (*M_RT_* = 1121 ms, *SD_RT_* = 301 ms, *Min_RT_* = 315 ms, *Max_RT_* = 1496) skewed toward the 1500-ms cutoff. [↑](#footnote-ref-1)
